# Enhanced performance of molecular electrocatalysts for CO_2_ reduction in a flow cell following K^+^ addition

**DOI:** 10.1126/sciadv.adh9986

**Published:** 2023-11-08

**Authors:** Shunsuke Sato, Keita Sekizawa, Soichi Shirai, Naonari Sakamoto, Takeshi Morikawa

**Affiliations:** Toyota Central Research and Development Laboratories, Incorporated, Nagakute, Aichi 480-1192, Japan.

## Abstract

Electrocatalytic CO_2_ reduction is a key aspect of artificial photosynthesis systems designed to produce fuels. Although some molecular catalysts have good performance for CO_2_ reduction, these compounds also suffer from poor durability and energy efficiency. The present work demonstrates the improved CO_2_ reduction activity exhibited by molecular catalysts in a flow cell. These catalysts were composed of a cobalt-tetrapyridino-porphyrazine complex supported on carbon black together with potassium salt and were both stable and efficient. These systems were found to promote electrocatalytic CO_2_ reduction with a current density of 100 mA/cm^2^ and generated CO over at least 1 week with a selectivity of approximately 95%. The optimal catalyst gave a turnover number of 3,800,000 and an energy conversion efficiency of more than 62% even at 200 mA/cm^2^.

## Teaser

A potassium salt in a catalytic layer enhanced the activity of electrocatalytic CO_2_ conversion of molecular catalysts.

## INTRODUCTION

The development of photocatalytic systems capable of synthesizing useful organic compounds by reducing carbon dioxide (CO_2_) under sunlight is an increasingly important research area that addresses both global warming and fossil fuel shortages. However, the direct solar-powered reduction of CO_2_ to organic chemicals using electrons and protons extracted from water, mimicking photosynthesis in plants, is much more difficult than H_2_ generation via water splitting. Recently, solar-driven CO_2_ reduction with more than 10% solar conversion efficiency was reported, using a solar cell incorporating electrocatalysts such as Ag metal (*1*, *2*). The high solar conversion efficiency of this device can be partly attributed to the superior electrocatalytic activity of the Ag catalyst. This result indicates that the development of electrocatalytic CO_2_ reduction catalysts will play a key role in realizing artificial photosynthesis systems.

Electrocatalytic CO_2_ reduction can be conducted using metal or molecular catalysts (that is, metal complexes) under specific electrical bias conditions. These materials require the application of a large electrical potential to achieve catalytic CO_2_ reduction because the first step in CO_2_ conversion is the formation of a CO_2_•^−^ radical anion intermediate during single-electron reduction (*3*). The electrocatalysts that are currently used can facilitate proton-coupled multi-electron reactions [for example, CO_2_ + 2H^+^ + 2e^−^ → CO + H_2_O, −0.11 V versus reversible hydrogen electrode (RHE)] that require lower potentials than single-electron reactions (*4*). However, many CO_2_ reduction catalysts have deficiencies such as low product selectivity in the presence of water due to the preferential formation of H_2_ at 0.0 V (versus RHE). In contrast, metals such as Au and Ag can act as electrocatalysts for selective CO_2_ reduction in NaHCO_3_ solutions but the associated overpotentials are high (greater than 600 mV) (*5*). The market prices of certain hydrocarbon products (including CH_4_, MeOH, and C_2_H_4_) are notably lower than those of other fine chemicals (*6*, *7*). Therefore, the development of a carbon-neutral society based partly on electrocatalytic CO_2_ reduction will require the development of systems operating at low overpotentials (that is, low cell voltages) while exhibiting increased current densities, good selectivity, and high durability. Recently, the current densities associated with CO_2_ reduction have been markedly increased through the use of gas diffusion layer electrolyzers (*8*–*12*). Values in excess of several hundreds of milliamperes per square centimeter have been reported based on incorporating metal catalysts such as Au, Ag, and Cu. Despite this, CO_2_ reduction catalysts still require improvement in several areas. As an example, because of the low mass-based catalytic activity during CO_2_ reduction provided by present-day materials, relatively large masses of noble metals must be used. In addition, the electrical-to-chemical conversion efficiencies obtainable during CO_2_ reduction in conjunction with water oxidation [meaning the energy conversion efficiency (*EE*)] are presently insufficient even when using noble metals (see table S1). One of the reviews has reported that the *EE* of electrocatalytic CO_2_ conversion must be greater than 60% at a current density of several hundreds of milliamperes per square centimeter to allow this process to become competitive with fossil fuel prices (*6*). However, the *EE* of CO_2_ electrolysis at room temperature was typically less than 40% over long-term experiments (*13*). The cost of electrolyzers is also an important aspect of the economic viability of this process, and so there is a need for high-efficiency, non-noble catalysts for CO_2_ reduction that meet the criteria described above.

A number of molecular catalysts have also been used in gas diffusion layer electrolyzers (*14*–*18*). Although these compounds have exhibited high current density values during CO_2_ reduction, there are associated deficits, such as low durability and poor *EE* (meaning high cell voltages or overpotentials). The poor durability of these materials is especially concerning (*19*). As an example, it has been reported that a cobalt phthalocyanine [Co(Pc)] catalyst with phenol as an additive produced a good current density of 150 mA/cm^2^ during CO_2_ reduction at approximately −2.3 V in a zero-gap membrane electrode assembly (MEA) cell. Although this device was able to promote the CO_2_ reduction reaction without resistance compensation, the catalytic activity of the Co(Pc) was maintained for less than several hours (at which point the current density decreased below 100 mA/cm^2^) and a high cell voltage was required (*14*). The CO_2_ reduction potentials and catalytic activities of molecular catalysts can be adjusted by changing the ligands and substituents in the complex (*19*), so it may be possible to develop materials based on tuning the molecular design and reaction environment (*20*). The goal of such work would be to achieve the required high *EE* along with suitable levels of current density and sufficient durability (*6*, *7*).

The present work demonstrates electrocatalytic CO_2_ reduction using an MEA cell including a cathode comprising cobalt-tetrapyridino-porphyrazine [Co(PyPc)] with potassium triflate (KOtf) as an additive on carbon-based support, together with nickel foam incorporating iron and nickel oxide catalysts as the anode and an anion exchange membrane (AEM). The electrocatalytic activity of Co(PyPc) was also markedly improved by adding K^+^ cations to the carbon substrate. This work therefore developed an electrocatalytic CO_2_ conversion system capable of producing CO from CO_2_ with high selectivity (approximately 95%) together with a low cell voltage (approximately −1.9 V), suitable *EE* (approximately 68%) and high durability (over 1 week under 100 mA/cm^2^) with a turnover number (TON) on the order of 3,800,000.

## RESULTS AND DISCUSSION

### Electrocatalytic CO_2_ reduction using an MEA cell including potassium salt in a catalyst layer

**Fig. 1.**
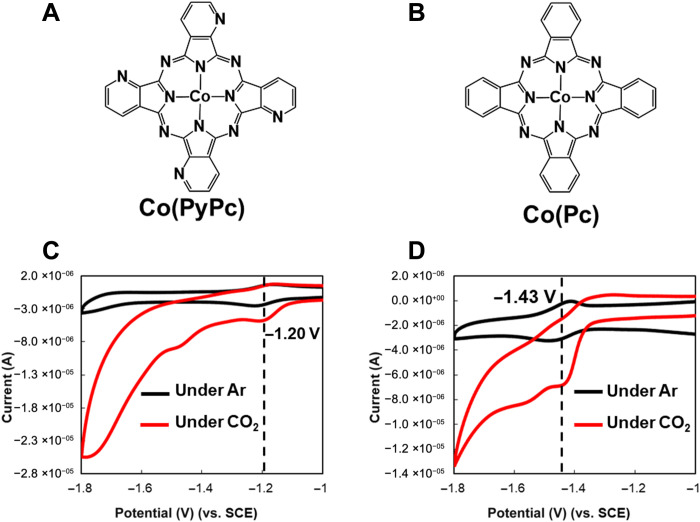
Molecular structures and cyclic voltammograms. Molecular structures of the (**A**) Co(PyPc) and (**B**) Co(Pc) electrocatalysts. Cyclic voltammograms obtained from (**C**) Co(PyPc) and (**D**) Co(Pc) in dimethyl formamide containing 0.1 M NEt_4_^+^BF_4_^−^ under Ar (black) and under CO_2_ (red). Measurements were conducted using a glassy carbon working electrode, a Pt counter electrode, and an Ag/AgNO_3_ reference electrode at a scan rate of 50 mVs^−1^.

These Co-based molecular catalysts were synthesized according to a previously reported method (*21*) and had the molecular structures shown in Fig. 1 (A and B). Figure 1 (C and D) presents cyclic voltammetry (CV) data obtained from Co(PyPc) and Co(Pc) in a dimethyl formamide (DMF) solution under Ar or CO_2_ atmospheres. The lowest unoccupied molecular orbital (LUMO) potential of Co(PyPc) was found to be lower than that of Co(Pc) by approximately 200 mV, and the former material generated a catalytic current under CO_2_. Density functional theory (DFT) calculations also indicated that Co(PyPc) catalysts should exhibit lower LUMO potentials than Co(Pc) (*22*). These results demonstrate that CO_2_ reduction using an MEA cell should proceed at a lower voltage when using Co(PyPc) instead of Co(Pc). In general, the CO_2_ reduction activity of a molecular catalyst having a lower LUMO potential is decreased because the compound does not readily react with CO_2_ (*17*, *20*, *23*). For this reason, the majority of research in this field has not examined molecular catalysts having lower LUMO potentials. However, the present work confirms that the synergistic effect of carbon support and K^+^ cations improves the CO_2_ reduction activity of molecular catalysts with lower LUMO potentials (*20*). We also reported that the CO_2_ reduction performance was markedly decreased by the absence of K^+^ cations in an aqueous solution using some metal complex catalysts (fig. S1). These results also suggest that K^+^ cations have a very important role in the CO_2_ reduction reaction. Therefore, it is possible that the electrocatalytic activity of CO_2_ reduction using Co complex catalysts can be improved by adding K^+^ cations in an MEA cell because only CO_2_ gas is provided near the catalysts in an MEA cell, that is, there is almost no K^+^ cation near the catalyst.

The present MEA cell consisted of a cathode, anode, AEM, and a cell plate with a flow system and Teflon spacer (fig. S2). Co(PyPc) supported on carbon powder, KOtf, and Nafion was drop-cast onto AvCarb gas diffusion paper to fabricate the cathode [referred to here as Co(PyPc) + K/C]. Transmission electron microscopy (TEM) images of the Co(PyPc) on carbon powder are presented in fig. S3. A Ni-doped β-FeOOH catalyst (*24*, *25*) on a Ni foam (Ni + Fe/Ni) was used as the anode. The catalytic areas of the cathode and anode were each 1.13 cm^2^. Wet CO_2_ was fed to the cathode at 100 standard cubic centimeters per minute (sccm), while a recirculated 1 M KOH solution was fed to the anode at a flow rate of 100 ml/min. The cell was equipped with an autosampler for in situ measurements and was directly connected to a gas chromatograph (GC) to allow the analysis of CO and H_2_ generated as reaction products. The electrocatalytic CO_2_ reduction trials in this cell were performed under constant current conditions over a time span of 2 hours, with the results shown in Table 1. The electrocatalytic activity during CO production throughout this 2-hour period, as indicated by current density, was found to be in the range of 10 to 200 mA/cm^2^. Note that, in trials without the Co(PyPc) catalyst, only H_2_ production was observed (fig. S4).

**Table 1.** Summary of the electrocatalytic CO_2_ reduction during −10 to − 200 mA/cm^2^. Summary of the results obtained from electrocatalytic CO_2_ reduction trials using the Co(PyPc) + K/C elect rode loaded with 0.06 mg of Co(PyPc) with the MEA cell for 2 hours at various constant current densities. All chronopotentiometry data are provided in figs. S29 and S30.

Current density (mA cm^−2^)	Cell voltage† (V)	*FE*(CO) (%)	Mass activity for CO* (mA mg^−1^)	EE* (%)
−10	−1.59	90.8 ± 4.4	151.3	76.4
−25	−1.71	94.3 ± 3.2	392.9	74.0
−50	−1.76	97.4 ± 1.3	811.7	74.0
−75	−1.82	96.8 ± 3.2	1210.0	71.2
−100	−1.89	98.2 ± 1.0	1636.7	69.5
−150	−2.04	92.3 ± 0.5	2307.5	60.7
−50†	−1.71	95.5 ± 0.5	191.9	74.8
−100†	−1.82	96.7 ± 2.9	402.9	71.2
−150†	−1.91	95.3 ± 2.1	595.6	66.8
−200†	−2.03	95.2 ± 3.6	793.3	62.8

*These data represent averages over the 2-hour reaction time span.

†In these trials, 0.24 mg Co(PyPc) was loaded on the electrode.

**Fig. 2.**
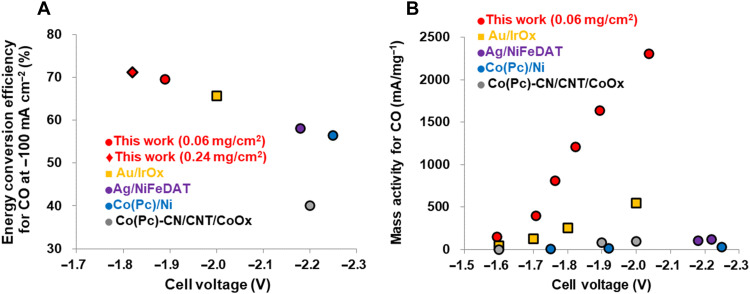
Comparisons of the catalytic activity for CO_2_ reduction. Comparisons of the results reported in this work (red) with the literature data [yellow (*9*), purple (*12*), blue (*14*), and gray (*15*)] in terms of (**A**) the *EE* during CO production at −100 mA/cm^2^ and (**B**) mass-based activity as a function of the cell potential without resistance compensation in conjunction with water oxidation using a KOH solution at room temperature.

The highest *EE* obtained from this device was approximately 76.4% at −1.59 V. Figure 2 summarizes the efficiencies observed during CO production trials using other catalysts for CO_2_ reduction with water oxidation at a current density of 100 mA/cm^2^. A cell voltage of approximately −1.89 V was obtained from an MEA cell containing the Co(PyPc) + K/C cathode [loaded with Co(PyPc) at 0.06 mg/cm^2^] with the Ni + Fe/Ni anode in conjunction with a current density of −100 mA/cm^2^ and a faradaic efficiency of 98.2 ± 1.0% during CO production [*FE*(CO)] and 69.5% *EE*. This MEA system, which uses only Earth-abundant elements such as Co, Fe, and Ni, exhibits superior performance (including a low cell voltage, high *EE*, and good mass-based activity) compared with Au and IrOx catalysts in MEA systems (for example, −100 mA/cm^2^ at −2.0 V using Au and IrOx) (*9*). In addition, the cell voltage during CO_2_ reduction could be lowered from −1.89 to −1.82 V by increasing the Co(PyPc) catalyst loading from 0.06 to 0.24 mg/cm^2^. This change also provided an *EE* of 71.2% at 100 mA/cm^2^. These results indicated that an enhanced current density together with high *EE* could be obtained simply by increasing Co(PyPc) in the device. The mass-based activity of the Co(PyPc) + K/C electrode was found to be approximately 2300 at a −2.04-V cell voltage, and so was on the order of five times greater than that reported for an Au catalyst at the same cell voltage (Fig. 2B). The mass-based activity of the present catalytic system was also approximately 130 times higher than that of the Co(Pc) catalyst used in prior research at the same cell voltage (*14*). To the best of our knowledge, this catalyst exhibited the highest mass-based activity during CO_2_ reduction, together with more than 60% *EE* using KOH solution (table S1).

**Fig. 3.**
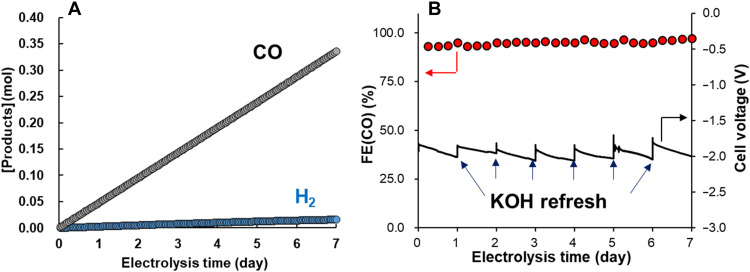
Long-term stability test at 100 mA/cm^2^. Electrocatalytic activity of the Co(PyPc) + K/C electrode using the MEA cell during long-term bulk electrolysis at −100 mA/cm^2^. (**A**) Moles of CO (black) and H_2_ (blue) produced using the Co(PyPc) + K/C electrode loaded with 0.05 mg of Co(PyPc) and (**B**) faradaic efficiency during CO production [*FE*(CO), red circles] and cell potential at a constant current density of −100 mA/cm^2^ (black line).

Figure 3A summarizes the electrocatalytic formation of CO over the Co(PyPc) + K/C using the MEA cell during a prolonged trial in which a reaction current of −100 mA/cm^2^ was obtained with cell voltages (Fig. 3B). The main product from this reaction was CO with an *FE*(CO) value of 95.0 ± 1.4% and the cell was able to demonstrate electrocatalytic activity for at least 7 days (Fig. 3, A and B) while generating approximately 0.34 mol of CO. Considering that approximately 78 nmol of Co(PyPc) was loaded on the AvCarb substrate, the turnover frequency (TOF) for CO production was approximately 6.4 s^−1^, while the TON was 3,859,745. In the case of the previous Co(Pc) catalyst, the TON was on the order of 4000 with a TOF of approximately 0.06 s^−1^ using an MEA cell at a current density of 100 mA/cm^2^. Our result showed that the improvement of TON for CO_2_ reduction was affected by K^+^ cations, which will be discussed later.

Although the cell voltage required for a current density of −100 mA/cm^2^ increased during the progression of the electrolysis, the original value could be almost fully recovered by refreshing the KOH solution (Fig. 3B). This effect occurred because the pH of the KOH solution decreased slightly from the initial value of 14.0 during the 24-hour electrolysis (*15*). The average cell voltage at 168 hours was approximately −1.95 V and the *EE* at this point was approximately 68%. The structure of the Co(PyPc) was assessed after 7 days of the reaction using x-ray photoelectron spectroscopy (XPS; fig. S5). These analyses indicated minimal change in the material after 168 hours of electrolysis. On the basis of this structural stability and the apparent recovery of the cell voltage after replacing the KOH solution, it is apparent that the present Co(PyPc)/C + K electrode would be expected to continuously generate CO over a period of 7 days. In general, MEA cells tend to suffer from the precipitation of carbonate salts at the cathode as a result of anolyte crossover (*26*, *27*). Therefore, it was anticipated that the addition of the KOtf salt to the catalytic layer on the cathode would lead to precipitation in the present system. However, salt precipitation was not observed in the flow channel after 7 days of electrolysis at a current density of −100 mA/cm^2^ nor after 120 hours at 150 mA/cm^2^ (fig. S6). Even so, as expected, the flow of gaseous CO_2_ was stopped by salt precipitation within several hours at −200 mA/cm^2^ (fig. S7). These results are in agreement with prior work (*27*) showing that salt precipitation is greatly affected by the amount of anolyte crossover and that K^+^ cations from this process are almost always consumed by salt precipitation. On the basis of these results and previous reports, we believe that the salt precipitation in the flow channel is not due to the presence of K^+^ cations but can be primarily attributed to OH^−^ anions resulting from anolyte crossover. Even when five times the amount of KOtf (5 mg) was added to the Co(PyPc) + K/C cathode, electrocatalytic CO_2_ reduction was maintained for 7 days (fig. S8) without any salt precipitation and if adding ca. 1 mg of KOH salt in the carbon layer, the salt precipitation has occurred within 2 hours (table S2). Even after a longer period of time (over 500 hours), no salt precipitation was observed in our system, and the catalytic activity remained stable (fig. S9).

### The potassium effect for CO_2_ reduction

In trials without KOtf addition, the Co(PyPc)/C electrode exhibited poor CO_2_ reduction activity along with low durability, and the requirement for a high cell voltage (fig. S10). The *FE*(CO) was also found to decrease from 95 to 50% within 4 hours and the cell voltage remained above 2.1 V in conjunction with a current density of −100 mA/cm^2^. These results indicated that K^+^ played a very important role in CO_2_ reduction over the Co(PyPc)/C in the MEA cell because the Co(PyPc) + K/C electrode was able to show continuous CO_2_ reduction activity for more than 168 hours with a cell voltage of less than −2.0 V after incorporating KOtf. This work confirmed that the durability of Co(PyPc) was increased by adding other salts such as potassium perfluoro-1-butanesulfonate (KC_4_F_9_Otf), sodium triflate (NaOtf), potassium bis(trifluoromethanesulfonyl)imide [K(Tf_2_N)], magnesium bis(trifluoromethanesulfonyl)imide [Mg(Tf_2_N)_2_], and cesium bis(trifluoromethanesulfonyl)imide [Cs(Tf_2_N)] (figs. S11 to S15), and the CO_2_ reduction activities of these systems were also maintained over a period of 24 hours. These results indicated that the CO_2_ reduction activity of the Co(PyPc) could be improved not only by the addition of KOtf but also by incorporating other alkali metal and alkaline earth metal salts (table S2). Although various alkali metal and alkaline earth metal salts were effective for the durability of CO_2_ reduction using a Co(PyPc) catalyst, potassium salts were more effective than other salts. Therefore, we mainly focused on the K^+^ effect for CO_2_ reduction. On the other hand, there is no effect for improving the CO_2_ reduction activity by adding K_2_CO_3_. Since the K_2_CO_3_ salt cannot be dissolved in catalysis ink [ethanol (EtOH) solution], K_2_CO_3_ crystals were only present on the carbon layer surface. It is well known that the precipitation of K_2_CO_3_ salts on the cathode surface is a result of anolyte crossover (*9*). Therefore, it can be said that the addition of K_2_CO_3_ reproduced almost the same condition as salting out due to anolyte crossover. This result clearly showed that there is no effect for improving the CO_2_ reduction activity by K_2_CO_3_ crystals “on” the carbon layer and anolyte crossover cannot provide the K^+^ source for improving the CO_2_ reduction activity. Therefore, it is assumed that the state in which K^+^ cations are incorporated into the carbon layer is important for CO_2_ reduction activity. In our gas diffusion electrode (GDE), we confirmed from cross-sectional scanning electron microscopy (SEM) and energy-dispersive x-ray (EDX) that K^+^ cations penetrate into the carbon layer (figs. S16 and S17). If the effect of the presence of K^+^ cations in the carbon layer is essential to improve CO_2_ reduction performance, then the same effect should be observed not only in MEA but also in the aqueous solution. The carbon electrode with Co(PyPc) was used for the CO_2_ reduction reaction using a three-electrode system in an aqueous solution in the absence of K^+^ cations (fig. S18). As in previous reports, hydrogen was the main product in aqueous solution without K^+^ cations. On the other hand, when 1 mg of KOtf salt was added to the carbon layer, CO became the main product. It is also clear that the presence of K salt in the carbon layer is important even in an aqueous solution since the addition of the same amount of K salt to the solution had no effect. This effect was confirmed not only for Co(PyPc) but also for Co tetraphenylporphyrin [Co(TPP)] as reported in the previous report (fig. S19). These results clearly indicated that the CO_2_ reduction performance can be improved by the presence of K^+^ cations in the carbon layer.

**Fig. 4.**
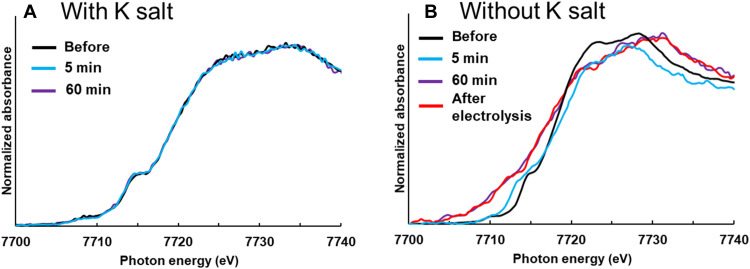
XANES spectra of Co catalyst with or without K salt. In situ XANES spectra of (**A**) Co(PyPc) + K/C and (**B**) Co(PyPc)/C electrodes at −1.4 V (versus Ag/AgCl) under CO_2_ [before electrolysis (black), 5-min electrolysis (blue), 60-min electrolysis (purple), and after electrolysis (red)].

Our prior work demonstrated that the CO_2_ reduction activities of catalysts made from Mn-, Co-, and Re-based complexes in aqueous solutions could be enhanced by a synergistic effect obtained by incorporating both K^+^ and carbon (fig. S1) (*20*). This previous research showed that K^+^ decreases the activation energy required for CO_2_ coordinated complex by interacting with CO_2_. The reaction mechanism for CO_2_ reduction using Co(Pc) has been reported (*12*, *28*), and it reported that the CO_2_ adsorption onto the Co center is the most energetically unfavorable during the reaction cycle. Therefore, it can be concluded that the overpotential for CO_2_ reduction in the MEA cell was decreased by the K^+^ effect on the CO_2_ adsorption onto the Co center. The K^+^ effect for improving the durability was followed by in situ x-ray absorption near edge structure (XANES) spectra (Fig. 4). This result clearly showed that the potassium salt in the catalyst layer prevented structural changes in the Co(PyPc) catalyst during electrolysis. If there is no potassium salt in the catalytic layer, then it turns into a metal-like structure. We confirmed whether the effect of the K^+^ effect could be applied to other catalysts. As a result, the CO_2_ reduction activity of the Co(Pc) was also improved by adding KOtf (fig. S20). In previous research, the Co(Pc) catalyst was deactivated within several hours at a current density of −100 mA/cm^2^ (*14*), while the present work showed that adding KOtf allowed CO production to persist for 24 hours with a reaction current of −100 mA/cm^2^. The cell voltage was also decreased from approximately −2.7 to −2.1 V even with small amounts of Co(Pc) (0.18 mg/cm^2^) loaded on the carbon layer. Recently, the mechanism by which CoTPP is deactivated during electrolytic CO_2_ reduction was established by experimental work and DFT calculations (*29*). One of the main reasons for the deactivation of Co(TPP) was found to be the off-center binding of CO_2_ on the complex structure. The off-center binding of CO_2_ via carbon atoms is thermodynamically favored over the adsorption of CO_2_ on the Co atom of the complex following two-electron reduction. It is possible that the Co(PyPc) catalyst was also deactivated via an off-center binding structure because tetraphenylporphyrin and phthalocyanine are very similar groups comprising tetrapyrrole macrocycles. For this reason, the DFT calculations were performed for Co(PyPc) with CO_2_ as with the prior work (*29*). In the case of the Co(PyPc) catalyst, the off-center binding of CO_2_ was also more stable than the adsorption of CO_2_ on the Co atom of a two-electron–reduced species (fig. S21). However, these same calculations confirmed that the adsorption of CO_2_ on the Co atoms was preferred following the addition of K^+^ (fig. S21, red line). The Co(Pc) catalyst also showed similar DFT results to the Co(PyPc) (fig. S22). Therefore, it appears that decomposition resulting from the off-center binding of CO_2_ can be prevented by adding a K^+^ salt to the catalyst layer in the MEA cell because the adsorption of CO_2_-K^+^ on the Co atom is thermodynamically favored over the off-center binding of CO_2_ structure. These results indicated that the presence of K^+^ and carbon provided a synergistic effect that improved the durability of the material while also decreasing the cell voltage required for CO_2_ reduction in an MEA cell with metal complex catalysts. Since the other alkali metal and alkaline earth metal cations also can adsorb CO_2_, it is assumed that the CO_2_ reduction reaction was improved by the same effect as K^+^.

### Isotope tracer experiment

The source of the carbon incorporated in the CO that was generated was assessed by isotope tracer analyses using ^13^CO_2_ with the Co(PyPc) + K/C electrode (fig. S23). CO with a mass/charge ratio (*m/z*) of 29 was found to be the main product, confirming that the CO detected in these electrocatalytic reactions over Co(PyPc) did not originate from other carbon sources, such as carbon black.

In conclusion, the potassium salt in the MEA cell prevented structural changes in Co complex catalysts and lowered the cell overpotential, leading to increased current densities and high durability together with low cell voltages. The cell voltage required for electrochemical CO_2_ reduction over the Co(PyPc) + K/C in the MEA cell was lowered to −1.59 V when using KOtf as an additive, and the long-term stability of the reductive reaction was improved such that the reaction proceeded for 7 days at 100 mA/cm^2^. As a result, the TON of CO production was more than 3,800,000. The Co(PyPc) + K/C in the MEA cell was also shown to function even at 200 mA/cm^2^ to produce CO. The effect by which K^+^ notably enhanced the CO_2_ reduction reaction rate along with the stability of the Co(PyPc)/C was also applicable to other catalysts such as Co(Pc). Furthermore, the CO_2_ reduction activity could be improved by adding other alkali metal and alkaline earth metal salts. This type of process could also be a pivotal aspect of developing robust flow cell systems for solar-driven CO_2_ reduction to produce organic chemicals using low input energy levels and only Earth-abundant materials. However, the anolyte crossover when using a KOH solution needs to be solved because it is one of the major factors in the durability and inhibition of current density improvement due to salt precipitation. This problem could be solved by using neutral solvents or by developing AEM. The Co(PyPc) can act as a catalyst for CO_2_ reduction even in neutral solvents (in KHCO_3_; fig. S24), but the anode electrode requires the use of IrOx catalyst because Ni + Fe/Ni catalysts do have not enough stability in the neutral solvents such as KHCO_3_. Although the Co(PyPc) + K/C electrode has the highest *EE* at 50 mA/cm^2^ in a KHCO_3_ solution than other catalysts (table S3), the *EE* was decreased than using the KOH solution. One of the main reasons that water oxidation activity was decreased at the neutral conditions (*30*). There are still remaining some problems using metal complex catalysts with MEA cells, but the present findings may suggest that such materials could generate organic compounds with commercially viable economics.

## MATERIALS AND METHODS

### Materials

Reagents, solvents, and Co(TPP) catalysts were purchased from FUJIFILM Wako Pure Chemical, Kanto Chemical, Tokyo Kasei, and Sigma-Aldrich and used without further purification. Carbon fiber paper (AVcarb 3520) was purchased from AvCarb Material Solutions.

### General procedures

The redox potentials of the complexes were determined in DMF containing tetraethylammonium tetrafluoroborate (NEt_4_^+^BF_4_^−^; 0.1 M) as a supporting electrolyte using CV with an ALS/CHI CHI-620 electrochemical analyzer. These trials used a glassy carbon disk working electrode (WE; 3-mm diameter), Ag/AgNO_3_ (0.1 M) reference electrode (RE), and Pt counter electrode (CE). The supporting electrolyte (NEt_4_^+^BF_4_^−^) was dried under vacuum at 373 K for 1 day before use. The molecular structures of the complexes were determined by time-of-flight mass spectrometry (Jeol JMS-T100LP) with methanol as the mobile phase. A potentiostat/galvanostat (SP-150, Bio-Logic Science Instruments) was used during the electrochemical catalytic reaction trials with the MEA cell system. Gaseous reaction products, such as CO and H_2_, were analyzed using a GC (Micro GC Fusion 3, INFICON). The amounts of each Co complex loaded on the carbon substrates were determined by inductively coupled plasma (ICP) analyses (Rigaku, CIROS 120 EOP). TEM images were obtained using a JEM-2100F instrument (Jeol) at an acceleration voltage of 200 kV. Co 2p and N 1s XPS data were acquired using a Quantera SXM instrument (ULVAC-PHI) with monochromated Al Kα radiation. AEM (thickness ca. 50 to 60 μm) and IrOx were synthesized according to a previously reported method (*31*).

### Fabrication of Co(PyPc) + K/C electrodes containing 0.05 or 0.06 mg of Co(PyPc)

The Co(PyPc) catalyst was synthesized according to a previously reported method (*21*). Electrodes were fabricated by adding 60 mg of carbon black (Vulcan XC 72R) to 40 ml of DMF followed by sonication for 30 min, after which 2 mg of Co(PyPc) was added followed by sonication for an additional 30 min. The sonicated solution was subsequently stirred for 24 hours at room temperature after which the resulting carbon black with Co(PyPc) was collected by filtration, washed with DMF and EtOH, and then dried in the air. The Co(PyPc) loading in the product was assessed by ICP and determined to be 0.02 mg of Co(PyPc)/1 mg of carbon. Subsequently, 10 mg of this carbon black with Co(PyPc), 1 mg of potassium triflate (KOtf), and 0.07 ml of a Nafion solution were added to 0.9 ml of EtOH. The resulting mixture was sonicated for 5 min and then agitated using a vortex mixer. A 0.05-ml quantity of this dispersion was then applied to an AVcarb 3520 substrate having a surface area of 1.13 cm^2^ and dried at 333 K for 5 min. This coating procedure was repeated five or six times so as to load either 0.05 or 0.06 mg of the Co(PyPc) onto the AVcarb, respectively. The resulting Co(PyPc) + K/C electrodes were allowed to stand in the dark at 333 K overnight. The electrode SEM images and EDX map data are shown in figs. S16 and S17.

### Fabrication of a Co(PyPc) + K/C electrode containing 0.24 mg of Co(PyPc)

The Co(PyPc) catalyst was synthesized according to a previously reported method (*21*). Electrodes were synthesized by adding 60 mg of carbon black (Vulcan XC 72R) to 40 ml of DMF followed by sonication for 30 min. Following this, 10 mg of the Co(PyPc) was added with sonication for a further 30 min, after which the dispersion was stirred for 24 hours at room temperature. The resulting carbon black with Co(PyPc) was collected by filtration, washed with DMF and EtOH, and then dried in the air. The Co(PyPc) loading was confirmed by ICP analysis to be 0.08 mg of Co(PyPc)/1 mg carbon.

Subsequently, 10 mg of the carbon black with Co(PyPc), 1 mg of KOtf, and 0.07 ml of a Nafion solution were added to 0.9 ml of EtOH and the mixture was sonicated for 5 min followed by agitation with a vortex mixer. A 0.05-ml quantity of this mixture was applied to an AVcarb 3520 substrate having a surface area of 1.13 cm^2^ and then dried at 333 K for 5 min. This procedure was repeated six times to deposit a total of 0.24 mg of the Co(PyPc) on the substrate and the resulting Co(PyPc) + K/C electrode was allowed to stand in the dark at 333 K overnight.

### Fabrication of a Co(PyPc)/C electrode

A 10-mg quantity of the carbon black with Co(PyPc) and 0.07 ml of a Nafion solution were added to 0.9 ml of EtOH and the dispersion was sonicated for 5 min and then agitated with a vortex mixer. A 0.05-ml portion of this mixture was dropped onto an AVcarb 3520 substrate having a surface area of 1.13 cm^2^ and then dried at 333 K for 5 min. This coating procedure was repeated six times to load a total of 0.06 mg of the Co(PyPc) on the substrate and the resulting Co(PyPc)/C electrode was then allowed to stand in the dark at 333 K overnight.

### Fabrication of a Co(Pc) + K/C electrode

The Co(Pc) catalyst was synthesized according to a previously reported method (*21*). The electrode was fabricated by adding 60 mg of carbon black (Vulcan XC 72R) to 40 ml of DMF followed by sonication for 30 min, after which 10 mg of the Co(Pc) was added followed by sonication for a further 30 min. The sonicated dispersion was stirred for 24 hours at room temperature, after which the carbon black with Co(Pc) was collected by filtration, washed with DMF and EtOH, and then dried in the air. An ICP analysis confirmed that the catalyst loading was 0.06 mg of Co(Pc)/1 mg of carbon.

A 10-mg quantity of the carbon black with Co(Pc), 1 mg of KOtf, and 0.07 ml of a Nafion solution were added to 0.9 ml of EtOH and the mixture was sonicated for 5 min followed by agitation with a vortex mixer. A 0.05-ml portion of this mixture was subsequently dropped onto an AVcarb 3520 substrate (1.13 cm^2^) and dried at 333 K for 5 min. This coating procedure was repeated six times to deposit a total of 0.18 mg of the Co(Pc) on the AVcarb. The resulting Co(Pc) + K/C electrode was allowed to stand in the dark at 333 K overnight.

### Fabrication of a Co(PyPc) + KC_4_F_9_Otf/C electrode

A 10-mg quantity of the carbon black with Co(PyPc), 1 mg of KC_4_F_9_Otf, and 0.07 ml of a Nafion solution were added to 0.9 ml of EtOH and the dispersion was sonicated for 5 min and then agitated using a vortex mixer. A 0.05-ml quantity of this dispersion was subsequently dropped onto an AVcarb 3520 substrate (1.13 cm^2^) and then dried at 333 K for 5 min. This coating procedure was repeated six times to load a total of 0.06 mg of the Co(PyPc) on the AVcarb. The resulting Co(PyPc) + KC_4_F_9_Otf /C electrode was allowed to stand in the dark at 333 K overnight.

### Fabrication of a Co(PyPc) + NaOtf/C electrode

A 10-mg quantity of the carbon black with Co(PyPc), 1 mg of NaOtf, and 0.07 ml of a Nafion solution were added to 0.9 ml of EtOH and the dispersion was sonicated for 5 min and then agitated using a vortex mixer. A 0.05-ml quantity of this mixture was subsequently dropped onto an AVcarb 3520 substrate (1.13 cm^2^) and then dried at 333 K for 5 min. This coating procedure was repeated six times to load a total of 0.06 mg of the Co(PyPc) on the AVcarb. The resulting Co(PyPc) + NaOtf/C electrode was allowed to stand in the dark at 333 K overnight.

### Fabrication of a Co(PyPc) + K(Tf_2_N)/C electrode

A 10-mg quantity of the carbon black with Co(PyPc), 2 mg of K(Tf_2_N), and 0.07 ml of a Nafion solution were added to 0.9 ml of EtOH and the dispersion was sonicated for 5 min and then agitated using a vortex mixer. A 0.05-ml quantity of this mixture was subsequently dropped onto an AVcarb 3520 substrate (1.13 cm^2^) and then dried at 333 K for 5 min. This coating procedure was repeated six times to load a total of 0.06 mg of the Co(PyPc) on the AVcarb. The resulting Co(PyPc) + K(Tf_2_N)/C electrode was allowed to stand in the dark at 333 K overnight.

### Fabrication of a Co(PyPc) + Mg(Tf_2_N)_2_/C electrode

A 10-mg quantity of the carbon black with Co(PyPc), 2 mg of Mg(Tf_2_N)_2_, and Cs(Tf_2_N) and 0.07 ml of a Nafion solution were added to 0.9 ml of EtOH and the dispersion was sonicated for 5 min and then agitated using a vortex mixer. A 0.05-ml quantity of this mixture was subsequently dropped onto an AVcarb 3520 substrate (1.13 cm^2^) and then dried at 333 K for 5 min. This coating procedure was repeated six times to load a total of 0.06 mg of the Co(PyPc) on the AVcarb. The resulting Co(PyPc) + Mg(Tf_2_N)_2_/C electrode was allowed to stand in the dark at 333 K overnight.

### Fabrication of a Co(PyPc) + Cs(Tf_2_N)/C electrode

A 10-mg quantity of the carbon black with Co(PyPc), 2 mg of Cs(Tf_2_N), and 0.07 ml of a Nafion solution were added to 0.9 ml of EtOH and the dispersion was sonicated for 5 min and then agitated using a vortex mixer. A 0.05-ml quantity of this mixture was subsequently dropped onto an AVcarb 3520 substrate (1.13 cm^2^) and then dried at 333 K for 5 min. This coating procedure was repeated six times to load a total of 0.06 mg of the Co(PyPc) on the AVcarb. The resulting Co(PyPc) + Cs(Tf_2_N)/C electrode was allowed to stand in the dark at 333 K overnight.

### Fabrication of a Co(PyPc) + KOH/C electrode

A 10-mg quantity of the carbon black with Co(PyPc), 0.1 ml of EtOH including KOH solution (100 mg of KOH/10 ml of EtOH), and 0.07 ml of a Nafion solution were added to 0.8 ml of EtOH and the dispersion was sonicated for 5 min and then agitated using a vortex mixer. A 0.05-ml quantity of this mixture was subsequently dropped onto an AVcarb 3520 substrate (1.13 cm^2^) and then dried at 333 K for 5 min. This coating procedure was repeated six times to load a total of 0.06 mg of the Co(PyPc) on the AVcarb. The resulting Co(PyPc) + NaOtf/C electrode was allowed to stand in the dark at 333 K overnight.

### Fabrication of a Co(PyPc) + K_2_CO_3_/C electrode

A 10-mg quantity of the carbon black with Co(PyPc), 1 mg of potassium carbonate (K_2_CO_3_), and 0.07 ml of a Nafion solution were added to 0.9 ml of EtOH and the dispersion was sonicated for 5 min and then agitated using a vortex mixer (the K_2_CO_3_ salt cannot be dissolved in this solution). A 0.05-ml quantity of this mixture was subsequently dropped onto an AVcarb 3520 substrate (1.13 cm^2^) and then dried at 333 K for 5 min. This coating procedure was repeated six times to load a total of 0.06 mg of the Co(PyPc) on the AVcarb. The resulting Co(PyPc) + K_2_CO_3_/C electrode was allowed to stand in the dark at 333 K overnight.

### Fabrication of a Ni + Fe/Ni electrode

The Ni-doped β-FeOOH catalyst was synthesized according to a previously reported method (*24*), after which 10 ml of an aqueous dispersion solution of the Ni-doped β-FeOOH, 150 mg of NiCl_2_·6H_2_O, and 75 mg of FeCl_2_·4H_2_O was added to 10 ml of pure water. A section of Ni foam was dipped into the resulting mixture and then calcined at 423 K for 2 hours under the air. Last, pre-electrolysis was conducted at +0.60 V (versus Ag/AgCl) for 1 hour in a 1.0 M KOH solution, after which the Ni + Fe/Ni electrode was cut to provide a specimen with a surface area of 1.13 cm^2^.

### Fabrication of a Co(PyPc) electrode for a three-electrode system

Ten milligrams of the carbon black with Co(PyPc) [1 mg of KOtf for Co(PyPc) with K salt electrode] and 0.07 ml of a Nafion solution were added to 0.9 ml of EtOH and the mixture was sonicated for 5 min followed by agitation with a vortex mixer. A 0.05-ml quantity of this mixture was applied to an AVcarb 3520 substrate (the area of the AVcarb 3520 substrate was 1.0 cm × 2.0 cm) and then dried at 333 K for 5 min. This procedure was repeated six times to deposit a total of 0.24 mg of the Co(PyPc) on the substrate (the drop-cast area was ca. 1.0 cm × 1.0 cm) and the resulting Co(PyPc) electrode was allowed to stand in the dark at 333 K overnight. Copper wire was connected to the AVcarb 3520 substrate of the Co(PyPc) electrode using Cu tape, and connection points were covered with thermoplastics using a glue gun.

### Fabrication of a Co(TPP) electrode for a three-electrode system

The Co(TPP) (1.0 mg, 0.0013 mmol) [and added 1 mg of KOtf for Co(TPP) with K salt electrode] dissolved in 1.0 ml of EtOH solution (0.05 ml) was dropped onto the AVcarb 3520 substrate (the drop-cast area was ca. 1.0 cm × 1.0 cm) and dried at 333 K for 5 to 10 min. The area of the AVcarb 3520 substrate was 1.0 cm × 2.0 cm. The coating procedure was repeated twice and the resulting Co(TPP) electrode was placed in the dark at 333 K overnight. Copper wire was connected to the AVcarb 3520 substrate of the Co(TPP) electrode using Cu tape, and connection points were covered with thermoplastics using a glue gun.

### Electrocatalytic reaction using MEA cell

The MEA cell electrolyzer (carbon dioxide electrolyzer purchased from Dioxide Materials) was composed of two flow plates, the sample and Ni + Fe/Ni or IrOx electrodes (each with a surface area of 1.13 cm^2^), Teflon spacers, and an AEM. A diagram of the MEA cell is provided in fig. S2. Wet CO_2_ was fed to the cathode at 100 sccm, while a recirculated 1 M KOH solution or 0.2 M KHCO_3_ was fed to the anode at a flow rate of 100 ml/min. The cell was equipped with an autosampler to allow for in situ measurements and was directly connected to a GC (Micro GC Fusion 3) for the analysis of CO and H_2_ using a thermal conductivity detector at 20-min intervals. A potentiostat/galvanostat (SP-150, Bio-Logic Science Instruments) was used for electrochemical measurements. All applied potentials and voltages were determined without iR compensation.

### Electrocatalytic reaction using a three-electrode system

Electrocatalytic reactions using a three-electrode system were performed at atmospheric pressure in a flow reactor with an SP-150. The Co(PyPc) or Co(TPP) cathodes (electrode size about 1.0 × 2.0 cm, reaction area about 1.0 cm^2^) were used as WEs. Ag/AgCl and platinum wire were used as the RE and CE, respectively. A Pyrex glass cell was used as the reactor vessel and 0.1 M K_2_B_4_O_7_ + 0.2 M K_2_SO_4_ solution or 0.1 M (NH_4_)_2_B_4_O_7_ + 0.2 M (NH_4_)_2_SO_4_ solution was used as the electrolyte. Then, CO_2_ gas was bubbled into the reactor for 60 min before the measurement and allowed to flow at 30 sccm during the measurement period. The cell was equipped with an autosampler to allow for in situ measurements and was directly connected to a GC (Micro GC Fusion 3) for the analysis of CO and H_2_ using a thermal conductivity detector at 20-min intervals. All applied potentials were determined without iR compensation.

### Computational details

The effects of the potassium cation (K^+^) on the CO_2_ adsorption to a Co(PyPc) were examined using quantum chemical calculations. Changes in free energy for the CO_2_ adsorption reactions (1) and (2) (Δ*G*_ads_) were calculated using the DFT method to focus on the effects of the K^+^ cation. In these reactions, adsorption of the carbon atom of the CO_2_ molecule to the central Co atom was assumed. The previous report (*20*) suggests that an electric double layer is formed on the carbon surface, indicating that the carbon surface is constantly negatively charged under the experimental conditions of use. Therefore, the total charge of the model was set to −2, regardless of the presence or absence of K^+^ ions. Henceforth, this type of adsorption is denoted as on-center binding.

$${\left[\mathrm{Co}(\mathrm{PyPc})\right]}^{2-}+{\mathrm{CO}}_{2}\to {\left[\mathrm{Co}(\mathrm{PyPc})({\mathrm{CO}}_{2})\right]}^{2-}$$ (1)

$${\left[\mathrm{Co}(\mathrm{PyPc})K\right]}^{2-}+{\mathrm{CO}}_{2}\to {\left[\mathrm{Co}(\mathrm{PyPc})({\mathrm{CO}}_{2})K\right]}^{2-}$$ (2)

Regarding the off-center binding of CO_2_, the following reaction (3) was examined.

$${\left[\mathrm{Co}(\mathrm{PyPc})\right]}^{-}+{\mathrm{CO}}_{2}\to [\mathrm{Co}{\left(\mathrm{PyPc})\{\mathrm{OC}(=O)\}\right]}^{2-}$$ (3)

The same reactions for a Co(Pc) were also assumed and calculated for comparison. The molecular geometrical structure of each species was optimized, and its Gibbs free energy at the optimized structure was obtained by carrying out vibrational analysis. The ωB97XD functional (*32*) was adopted for the DFT calculations. The basis set with relativistic compact effective potentials by Stevens *et al*. (*33*) was used for the Co atoms, while the 6-31G(d,p) basis set (*34*–*37*) was used for other elements. The solvent effects of water were incorporated into the calculations by adopting the polarizable continuum model (*38*–*43*). All the calculations were carried out using the Gaussian16 program (*44*).

The calculated Gibbs free energies and Δ*G*_ads_ values are summarized in table S4. The calculated Δ*G*_ads_ values for reaction (1) were positively high, indicating that the on-center binding is energetically unfavorable in the absence of the K^+^ cation for Co(PyPc). In contrast, Δ*G*_ads_ for reaction (2) is reduced from that of reaction (1). This result suggests that the K^+^ cation stabilizes the CO_2_ adsorbed structures to promote the on-center binding; the effect is more notable for highly reduced species. Figure S25 shows the Corey-Pauling-Koltun space-filling model of [Co(PyPc)(CO_2_)K]^2−^. The models show that the K^+^ cation interacts with not only the adsorbed CO_2_ molecule but also the phthalocyanine ring because of its large ionic radius (*45*), which is responsible for the stabilization of these species. This effect of the K^+^ cation is similar to that computationally analyzed in the CO_2_ reduction with the Mn-complex catalyst; the interaction of the K^+^ cation with the adsorbed CO_2_ molecule stabilizes the transition state and lowers the reaction barrier (*20*).

Meanwhile, the off-center binding of CO_2_ was also observed for [Co(PyPc){OC(=O)}]^2−^ (fig. S26); the CO_2_ molecule was adsorbed on one of the carbon atoms neighboring pyrrole nitrogen atoms, and the phthalocyanine ring was distorted from a planar structure. The calculated Δ*G*_ads_ for reaction (3) was −4.9 kJ/mol, which is reduced from Δ*G*_ads_ for reaction (1) (+39.2 kJ/mol) to a negative value. The results suggest that, in the absence of K^+^, the off-center binding of CO_2_ to a highly reduced Co(PyPc) is a preferable process to the on-center binding. Figure S25 shows that the K^+^ cation occupies one of the off-center-binding cites. This structural effect could block the off-center binding process, which can be responsible for improving the material’s durability.

The results for Co(Pc) were generally similar to those for Co(PyPc). The on-center binding of CO_2_ without K^+^ is energetically unfavorable, as suggested from the calculated Δ*G*_ads_ values for reaction (1). In contrast, the Δ*G*_ads_ values for reaction (2) was negative, suggesting that the K^+^ cation stabilizes the CO_2_ adsorbed structures to promote the on-center binding. The optimized structure for [Co(Pc)(CO_2_)K]^2−^ was quite similar to that for [Co(PyPc)(CO_2_)K]^2−^, suggesting the interactions of the K^+^ cation with both the adsorbed CO_2_ and phthalocyanine ring (fig. S27). The calculated Δ*G*_ads_ of reaction (3) was −27.3 kJ/mol, suggesting that the off-center binding of CO_2_ to [Co(PyPc){OC(=O)}]^2−^ (fig. S28) is a preferable process to the on-center binding in the absence of K^+^.

The Δ*G*_ads_ values for the reactions with Co(Pc) were generally lower than those for Co(PyPc). This trend can be attributed to the difference in the electron-donating property. The number of nitrogen atoms in the Pc ring is less than that of the PyPc ring. Therefore, the electron-donating property of Co(Pc) is higher than that of Co(PyPc), which results in stronger binding of the CO_2_ molecule.

### In situ x-ray absorption spectroscopy

The chemical states of Co were evaluated by x-ray absorption fine structure (XAFS) spectroscopy using the quick-XAFS technique (*46*). In situ XAFS measurement was performed using a custom-designed two-compartment cell in a three-electrode configuration. A Co(PyPc) + K/C and Co(PyPc)/C electrode were used as the WE, a silver/silver chloride (Ag/AgCl) electrode (EC Frontier, RE-T14) as the RE, and a platinum foil (Pt-foil) electrode (Niraco, PT-353212, ϕ20 mm × 0.02 mm, 99.98%) as the CE. The electrolysis in situ XAFS cell for electrocatalytic CO_2_ reduction consisted of an anode and a cathode separated by an AEM (ASE, Astom Co., Ltd.), together with a CO_2_-saturated 0.5 M aqueous solution of KHCO_3_ as the electrolyte. The WE has a Kapton film window for fluorescence XAFS measurement. During the in situ XAFS measurement, CO_2_ continuously flowed into the WE (cathode) side of the cell. Applied potential was controlled by bi-potentiostat (model 2325, ALS) at −1.4 V (versus Ag/AgCl).

### Calculation of the faradaic efficiency and current density associated with CO production during the electroreduction of CO_2_ to CO

The *FE*(CO) was calculated as

FE(CO)(%)=2×n/F×Q×100 (4)

where *n* is the moles CO, *F* is Faraday’s constant (96,485 C mol^−1^), and *Q* is the amount of charge passing through the system. The current density associated with CO production (*J*co) was calculated as

Jco=current density×FE(CO) (5)

### Calculation of the energy conversion efficiency for the electroreduction of CO_2_ to CO

The energy conversion efficiency for the electroreduction of CO_2_ to CO was calculated as

$$\text{Energy conversion efficiency}\;(EE)=FE(\mathrm{CO})\times E\mathrm{cell}{}^{\circ }/E\mathrm{cell}$$ (6)

where *E*cell° is the thermodynamic equilibrium potential for the reaction from CO_2_ to CO (1.34 V) and *E*cell is the applied cell voltage.

## Supplementary Materials

### This PDF file includes:

Figs. S1 to S30 Tables S1 to S4 References
