# Supplementary material for: Enhanced performance of molecular electrocatalysts for CO2 reduction in a flow cell following K+ addition
Source: Sci Adv. 2023 Nov 8;9(45):eadh9986. doi: 10.1126/sciadv.adh9986 (PMC10631738; doi:10.1126/sciadv.adh9986)
Supplement: Supplementary file 1 — Figs. S1 to S30 Tables S1 to S4 References [file sciadv.adh9986_sm.pdf]

Science Advances
AAAS

Supplementary Materials for

Enhanced performance of molecular electrocatalysts for CO_2 reduction in a flow cell following K^+ addition

Shunsuke Sato *et al.*

Corresponding author: Shunsuke Sato, ssato@mosk.tytlabs.co.jp

Sci. Adv. **9**, eadh9986 (2023)
DOI: 10.1126/sciadv.adh9986

This PDF file includes:

Figs. S1 to S30
Tables S1 to S4
References

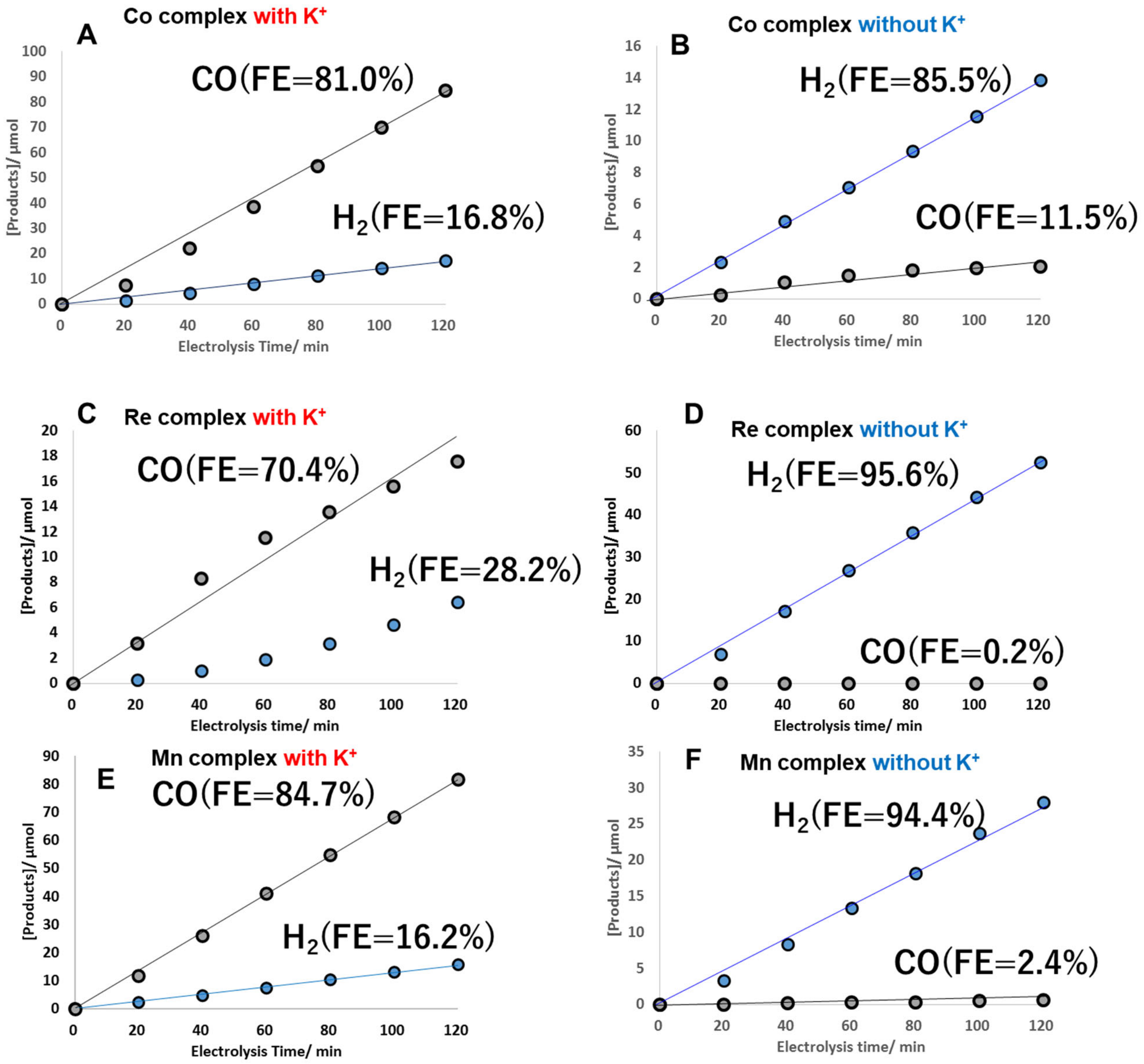

Fig. S1. Previous results of K effect for CO_2 reduction using metal complex catalysts. Electrocatalytic activity of Co complex ([Co(TPP)]), Re complex ([Re{4,4'-di(1H-pyrrolyl-3-propyl carbonate)-2,2'-bipyridine}$(CO)_3$(Cl)]) and Mn complex ([Mn{4,4'-di(1H-pyrrolyl-3-propyl carbonate)-2,2'-bipyridine}$(CO)_3$(MeCN)]) electrode indicating volume of carbon monoxide (black) and hydrogen (blue) produced. (A) Bulk electrolysis for 2 h at -0.59 V (*vs*. RHE) in a solution of CO_2-saturated 0.1 M $K_2B_4O_7$ + 0.2 M K_2SO_4 using Co complex electrode, (B) bulk electrolysis for 2 h at -0.59 V (*vs*. RHE) in a solution of CO_2-saturated 0.1 M $(NH_4)_2B_4O_7$ + 0.2 M $(NH_4)_2SO_4$ using Co complex electrode, (C) bulk electrolysis for 2 h at -0.59 V (*vs*. RHE) in a solution of CO_2-saturated 0.1 M $KHCO_3$ using Re complex electrode, (D) bulk electrolysis for 2 h at -0.59 V (*vs*. RHE) in a solution of CO_2-saturated 0.1 M NH_4CO_3 using Re complex electrode, (E) Bulk electrolysis for 2 h at -0.49 V (*vs*. RHE) in a solution of CO_2-saturated 0.1 M $K_2B_4O_7$ + 0.2 M K_2SO_4 using Mn complex electrode (F) bulk electrolysis for 2 h at -0.49 V (*vs*. RHE) in a solution of CO_2-saturated 0.1 M $(NH_4)_2B_4O_7$ + 0.2 M $(NH_4)_2SO_4$ using Mn complex electrode. Reprinted with permission from [ACS Catalysis 8, 4452-4458 (2018)]. Copyright 2023 American Chemical Society.

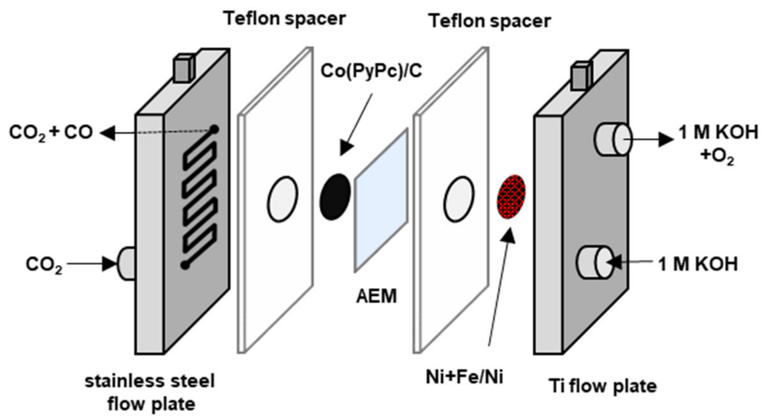

Fig. S2. Schematic illustration of reactor. Diagram of the MEA cell.

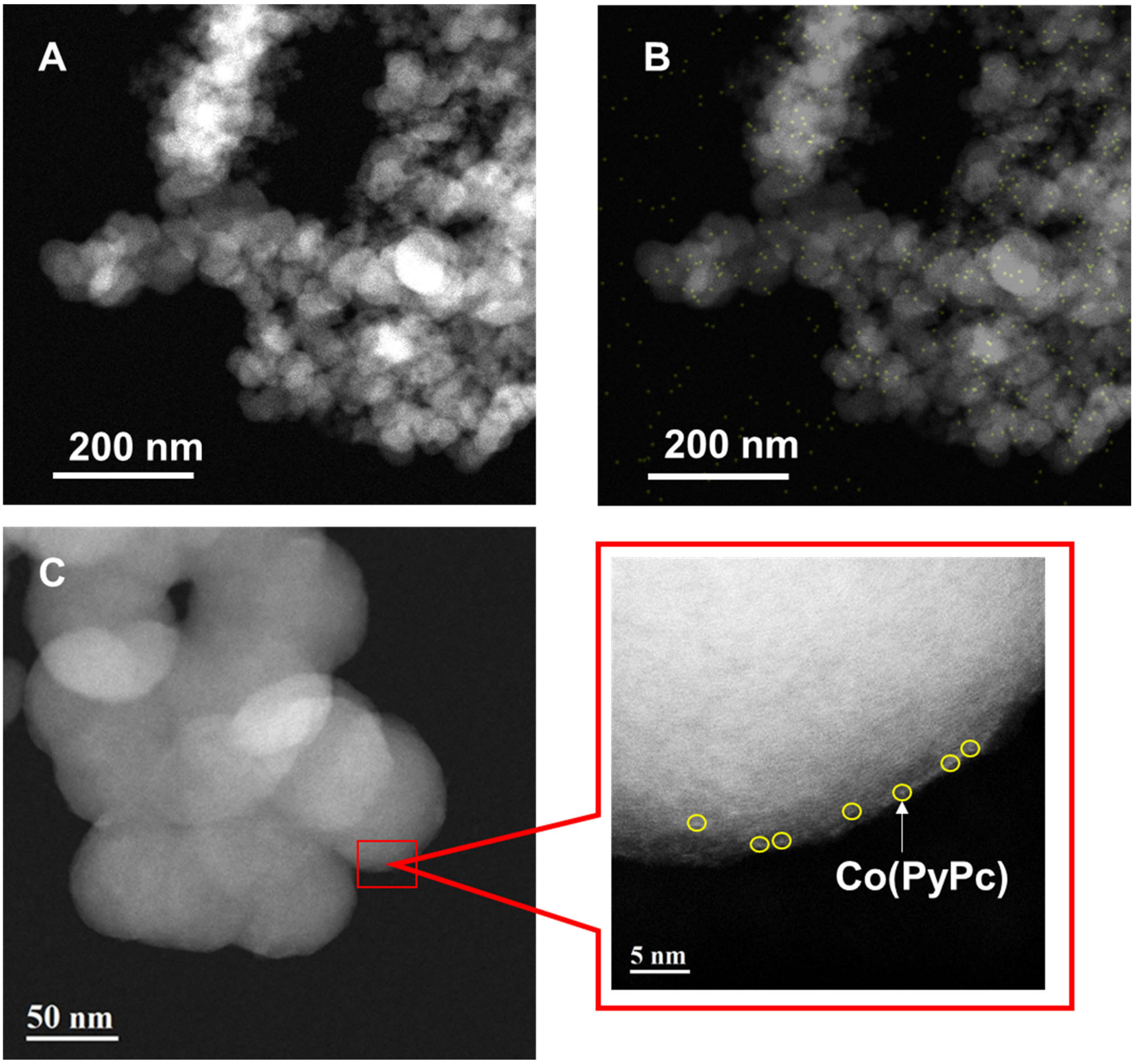

Fig. S3. TEM images of catalyst. TEM images of the carbon black with Co(PyPc). (A) A typical TEM image, (B) an EDS Co map (in which Co appears as green spots) and (C) a HADDF-STEM image of the carbon black with Co(PyPc). The bright spots in yellow circles in (C) correspond to the central Co atoms of Co(PyPc) complexes.

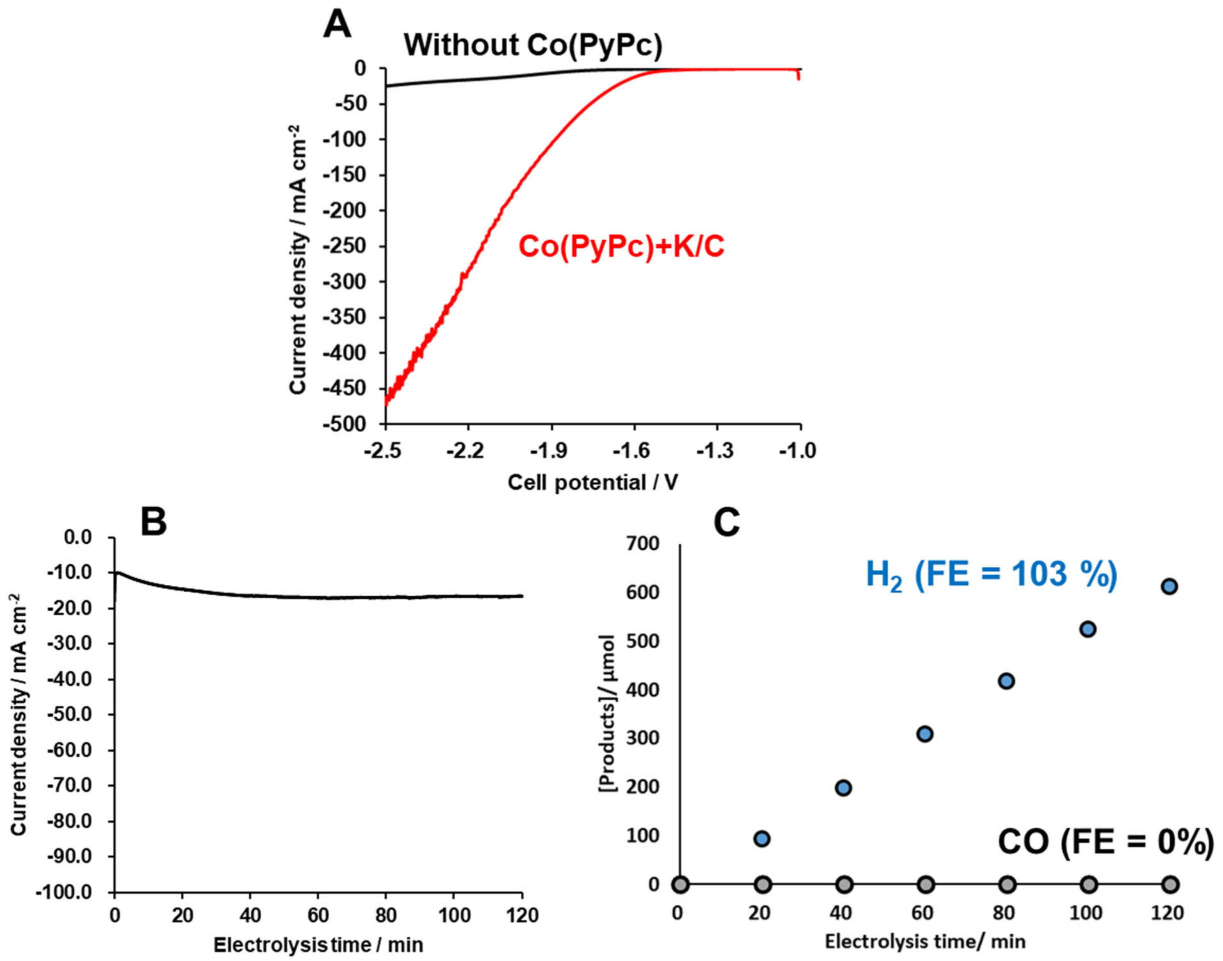

Fig. S4. Control experiments using without Co catalyst electrode. (A) Linear sweep voltammetry data for the K/C (black, without Co(PyPc)) and Co(PyPc)+K/C (red) electrocatalysts obtained using the MEA cell. (B) A chronoamperogram obtained from the K/C electrode during a 2 h electrolysis trial with a -1.9 V cell voltage. (C) Moles of CO (black) and hydrogen (blue) produced using the K/C electrode with the MEA cell, representing measures of the electrocatalytic activity of the material.

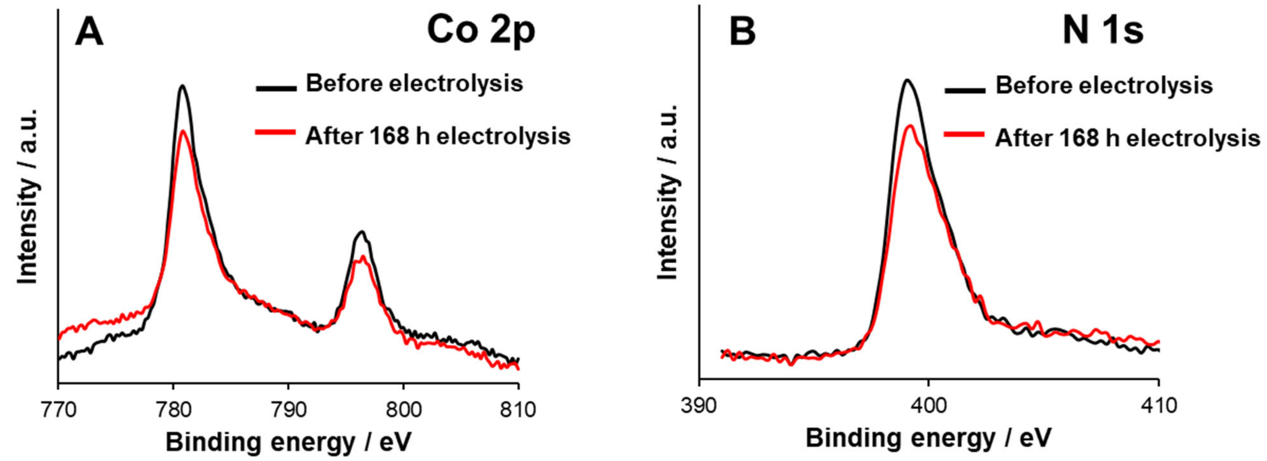

Fig. S5. XPS spectra of before and after electrodes. (A) Co 2p and (B) N 1s XPS data obtained from the Co(PyPc)+K/C electrode and after a 168 h electrolysis trial (TON = 3,859,745).

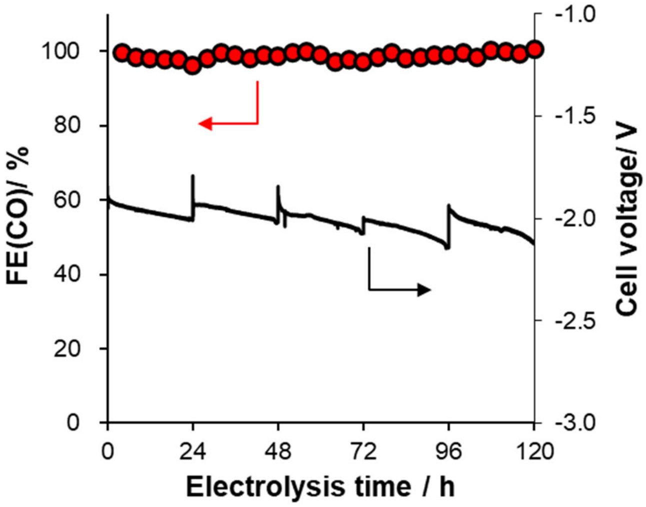

Fig. S6. Long-term bulk electrolysis at -150 mA/cm^2. Electrocatalytic activity of the Co(PyPc)+K/C electrode containing 0.24 mg Co(PyPc) using the MEA cell during long-term bulk electrolysis at -150 mA/cm^2 as shown by the Faradaic efficiency associated with CO production (FE(CO), red circles) and cell voltage at a constant current density of -150 mA/cm^2 (black line). During this trial, the KOH solution was refreshed after every 24 h of electrolysis.

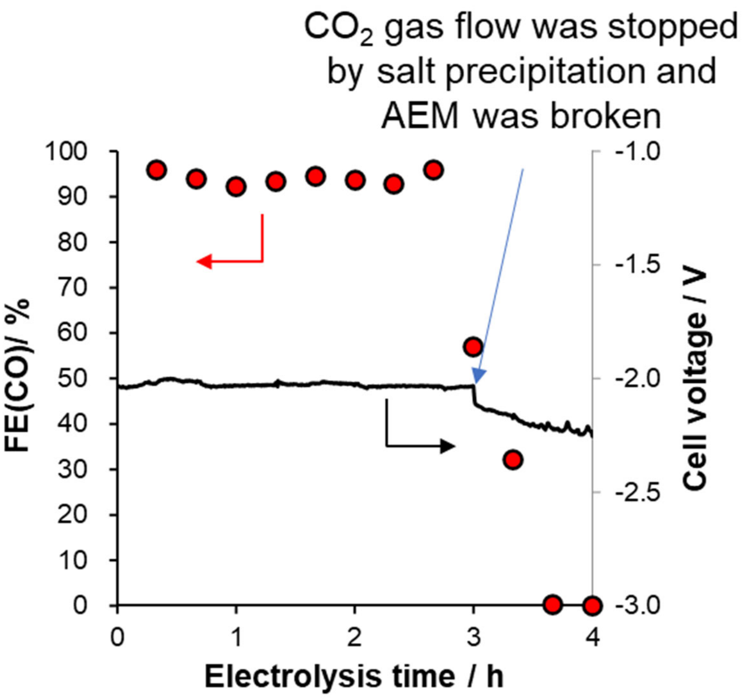

Fig. S7. Salt precipitation during electrolysis at -200 mA/cm^2. Electrocatalytic activity of the Co(PyPc)+K/C electrode containing 0.24 mg Co(PyPc) using the MEA cell during long-term bulk electrolysis at -200 mA/cm^2, as indicated by the Faradaic efficiency for CO production (FE(CO), red circles) and cell voltage at a constant current density of -200 mA/cm^2 (black line).

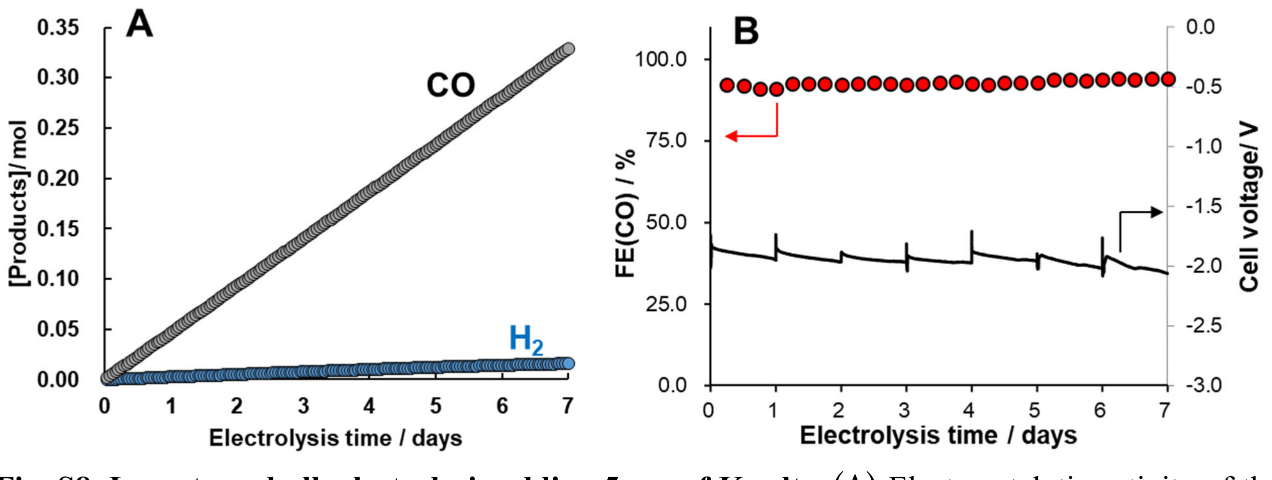

Fig. S8. Long-term bulk electrolysis adding 5 mg of K salts. (A) Electrocatalytic activity of the Co(PyPc)+K/C electrode containing 0.05 mg Co(PyPc) and 5 mg KOtf using the MEA cell during long-term bulk electrolysis at -100 mA/cm^2 as indicated by the moles of CO (black) and hydrogen (blue) produced. (B) Electrocatalytic activity of the Co(PyPc)+K/C electrode using the MEA cell during long-term bulk electrolysis at -100 mA/cm^2 as indicated by the Faradaic efficiency associated with CO production (FE(CO), red circles) and cell voltage at a constant current density of -100 mA/cm^2 (black line). During this trial, the KOH solution was refreshed after every 24 h of electrolysis.

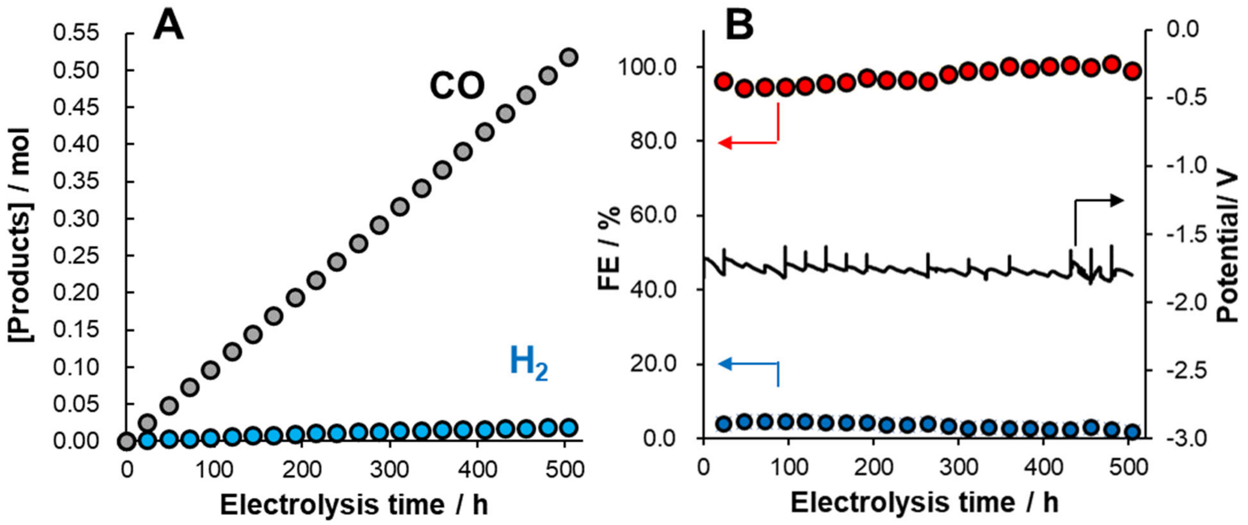

Fig. S9. Long-term bulk electrolysis for 500 h. (A) Electrocatalytic activity of the Co(PyPc)+K/C electrode containing 0.24 mg Co(PyPc) and 5 mg KOtf using the MEA cell during long-term bulk electrolysis at -50 mA/cm^2 as indicated by the moles of CO (black) and hydrogen (blue) produced. (B) Electrocatalytic activity of the Co(PyPc)+K/C electrode using the MEA cell during long-term bulk electrolysis at -50 mA/cm^2 as indicated by the Faradaic efficiency associated with CO production (FE(CO), red circles), H_2 production (FE(H_2), blue circles) and cell voltage at a constant current density of -50 mA/cm^2 (black line). During this trial, the KOH solution was refreshed after every 48 or 72 h of electrolysis.

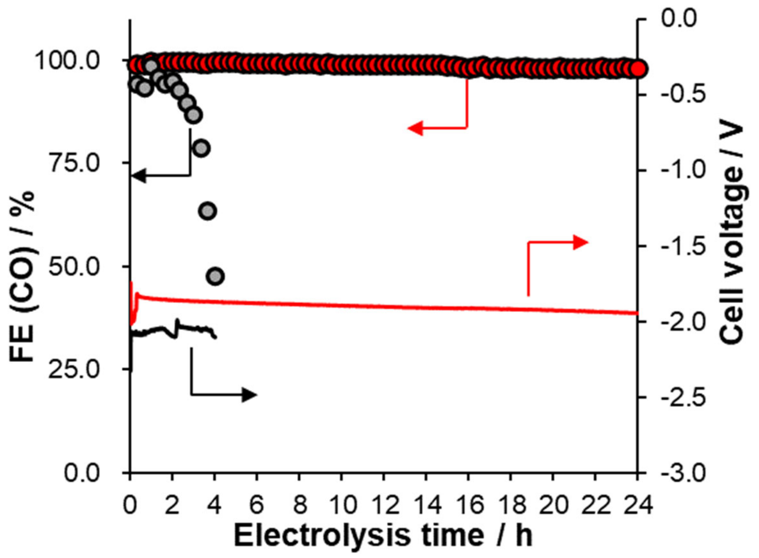

Fig. S10. Comparison of the K effect for CO_2 reduction using Co(PyPc). Electrocatalytic activities of the Co(PyPc)/C and Co(PyPc)+K/C electrodes each loaded with 0.06 mg Co(PyPc) using the MEA cell during long-term bulk electrolysis at -100 mA/cm^2. Faradaic efficiency for CO production using the Co(PyPc)+K/C (FE(CO), red circles) and Co(PyPc)/C (black circles) and cell voltages at a constant current density of -100 mA/cm^2 using the Co(PyPc)+K/C (red line) and Co(PyPc)/C (black line) are shown.

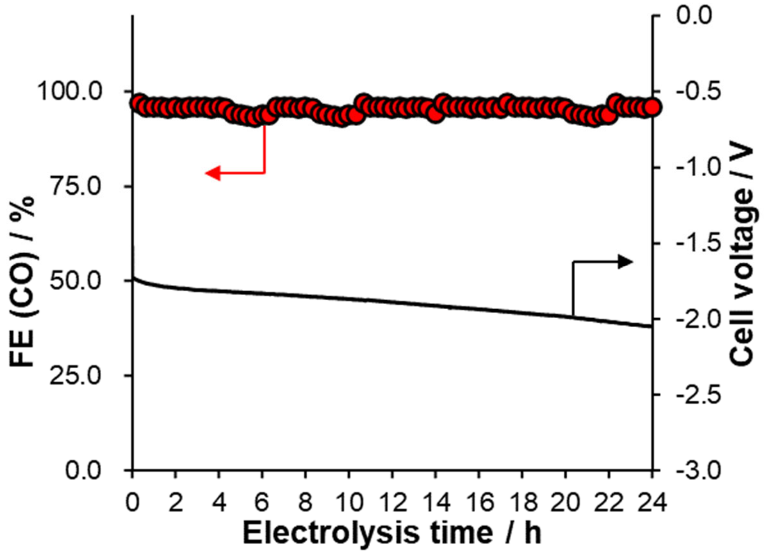

Fig. S11. Electrocatalytic CO_2 reduction adding KC_4F_9Otf salt. Electrocatalytic activity of the Co(PyPc) + KC_4F_9Otf/C electrode loaded with 0.06 mg Co(PyPc) using the MEA cell during long-term bulk electrolysis at -100 mA/cm^2, as indicated by the Faradaic efficiency for CO production (FE(CO), red circles) and cell voltage at a constant current density of -100 mA/cm^2 (black line).

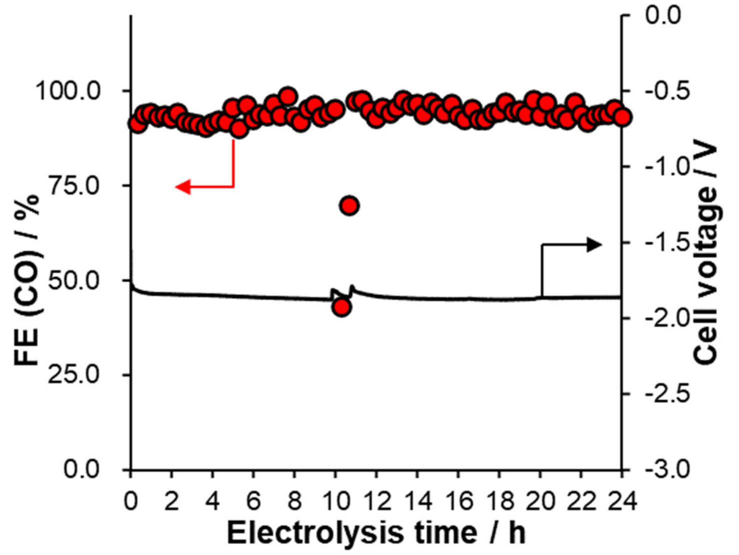

Fig. S12. Electrocatalytic CO_2 reduction adding NaOtf salt. Electrocatalytic activity of the Co(PyPc)+NaOtf/C electrode loaded with 0.06 mg Co(PyPc) using the MEA cell during long-term bulk electrolysis at -50 mA/cm^2, as indicated by the Faradaic efficiency for CO production (FE(CO), red circles) and cell voltage at a constant current density of -50 mA/cm^2 (black line).

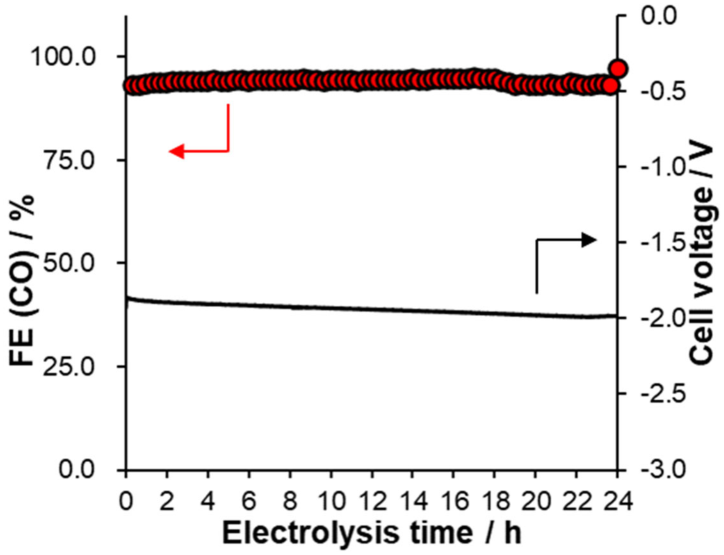

Fig. S13. Electrocatalytic CO_2 reduction adding K(Tf_2N) salt. Electrocatalytic activity of the Co(PyPc)+K(Tf_2N)/C electrode loaded with 0.06 mg Co(PyPc) using the MEA cell during long-term bulk electrolysis at -100 mA/cm^2, as indicated by the Faradaic efficiency for CO production (FE(CO), red circles) and cell voltage at a constant current density of -100 mA/cm^2 (black line).

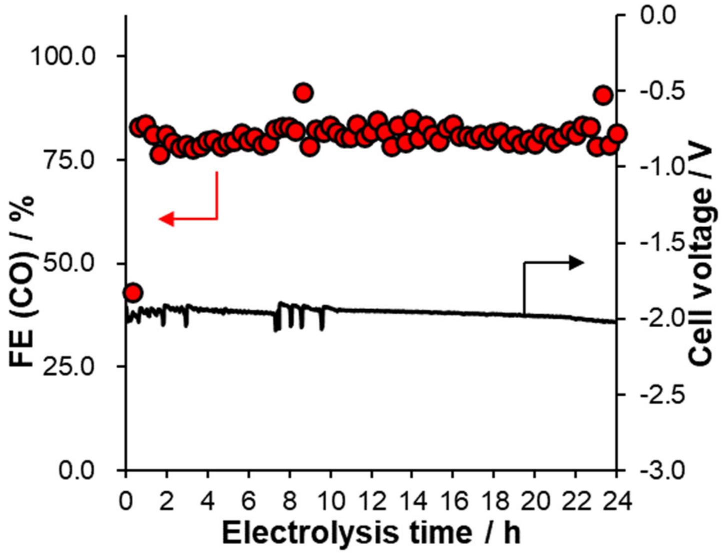

Fig. S14. Electrocatalytic CO_2 reduction adding Mg(Tf_2N)$_2$ salt. Electrocatalytic activity of the Co(PyPc)+Mg(Tf_2N)$_2$/C electrode loaded with 0.06 mg Co(PyPc) using the MEA cell during long-term bulk electrolysis at -100 mA/cm^2, as indicated by the Faradaic efficiency for CO production (FE(CO), red circles) and cell voltage at a constant current density of -100 mA/cm^2 (black line).

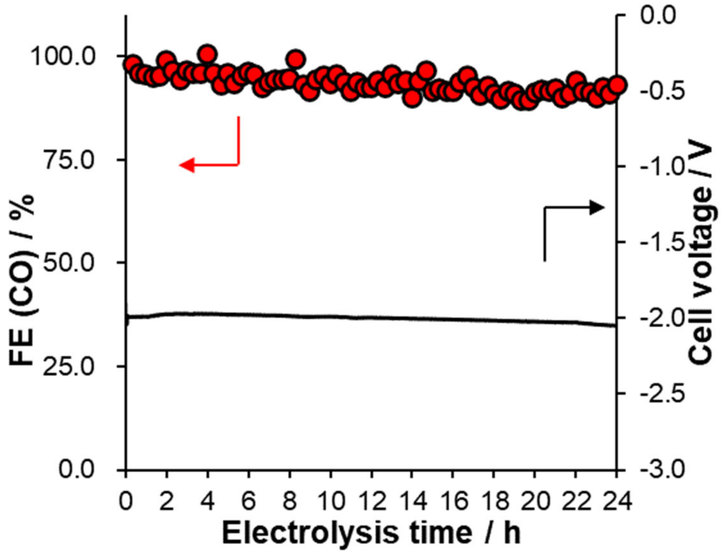

Fig. S15. Electrocatalytic CO_2 reduction adding Cs(Tf_2N) salt. Electrocatalytic activity of the Co(PyPc)+Cs(Tf_2N)/C electrode loaded with 0.06 mg Co(PyPc) using the MEA cell during long-term bulk electrolysis at -50 mA/cm^2, as indicated by the Faradaic efficiency for CO production (FE(CO), red circles) and cell voltage at a constant current density of -50 mA/cm^2 (black line).

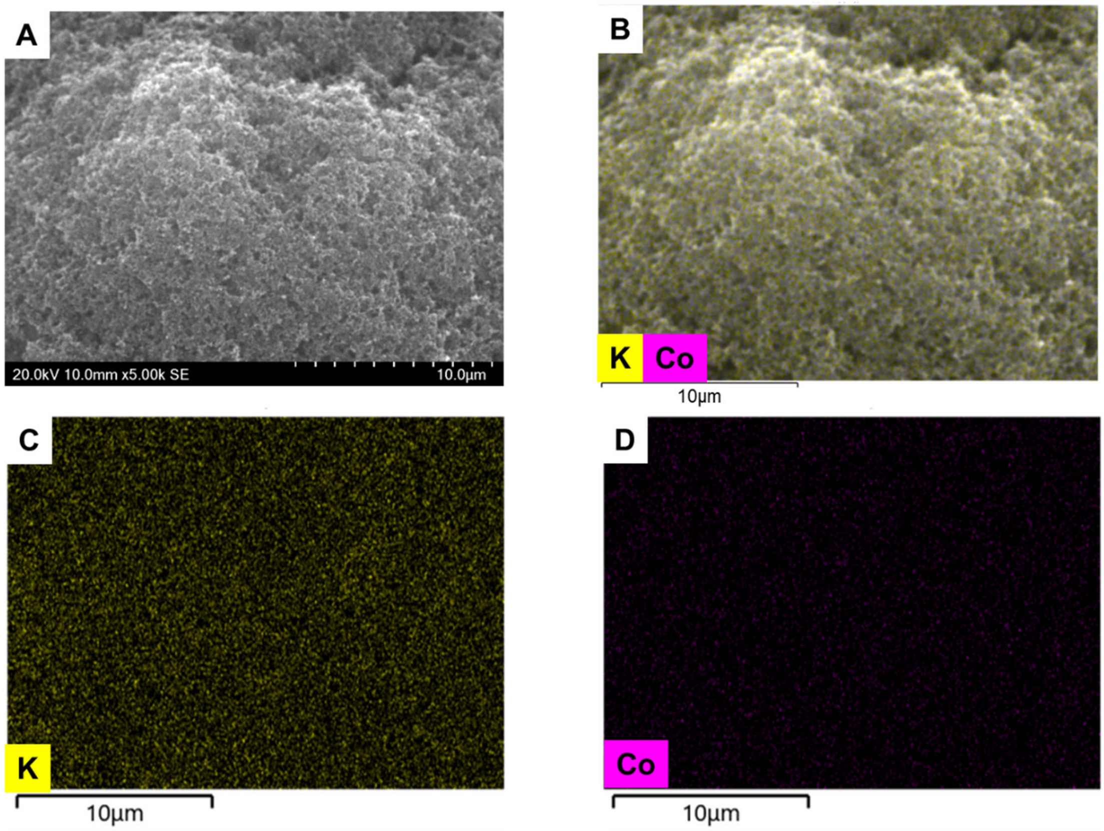

Fig. S16. SEM images of Co(PyPc)+K/C electrode. Scanning electron microscope images of the surface of (A) Co(PyPc)+K/C electrode. (B) An EDS Co and K map with SEM images (in which Co appears as purple spots and K appears as yellow spots), (C) an EDS K map (in which K appears as yellow spots) and (D) an EDS Co map (in which Co appears as purple spots). These images were obtained by using scanning electron microscope SU3500 (Hitachi High-Technologies Corporation).

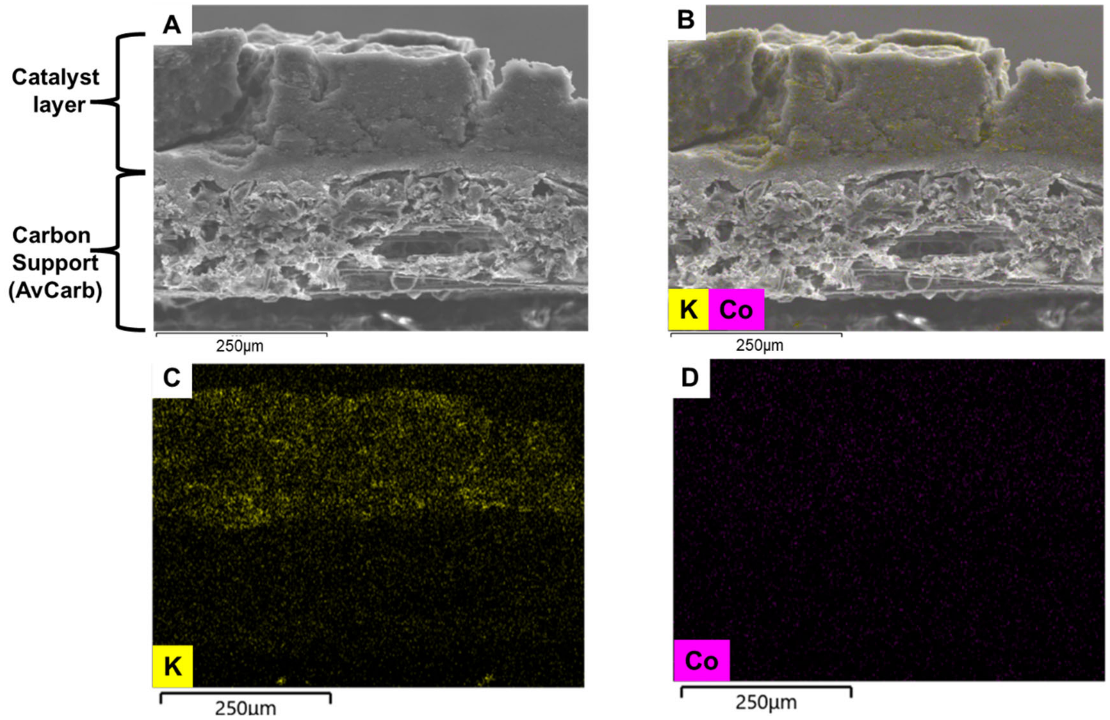

Fig. S17. SEM images of cross section of Co(PyPc)+K/C electrode. Scanning electron microscope images of the cross section of (A) Co(PyPc)+K/C electrode. (B) An EDS Co and K map with SEM images (in which Co appears as purple spots and K appears as yellow spots), (C) an EDS K map (in which K appears as yellow spots) and (D) an EDS Co map (in which Co appears as purple spots). These images were obtained by using scanning electron microscope SU3500 (Hitachi High-Technologies Corporation).

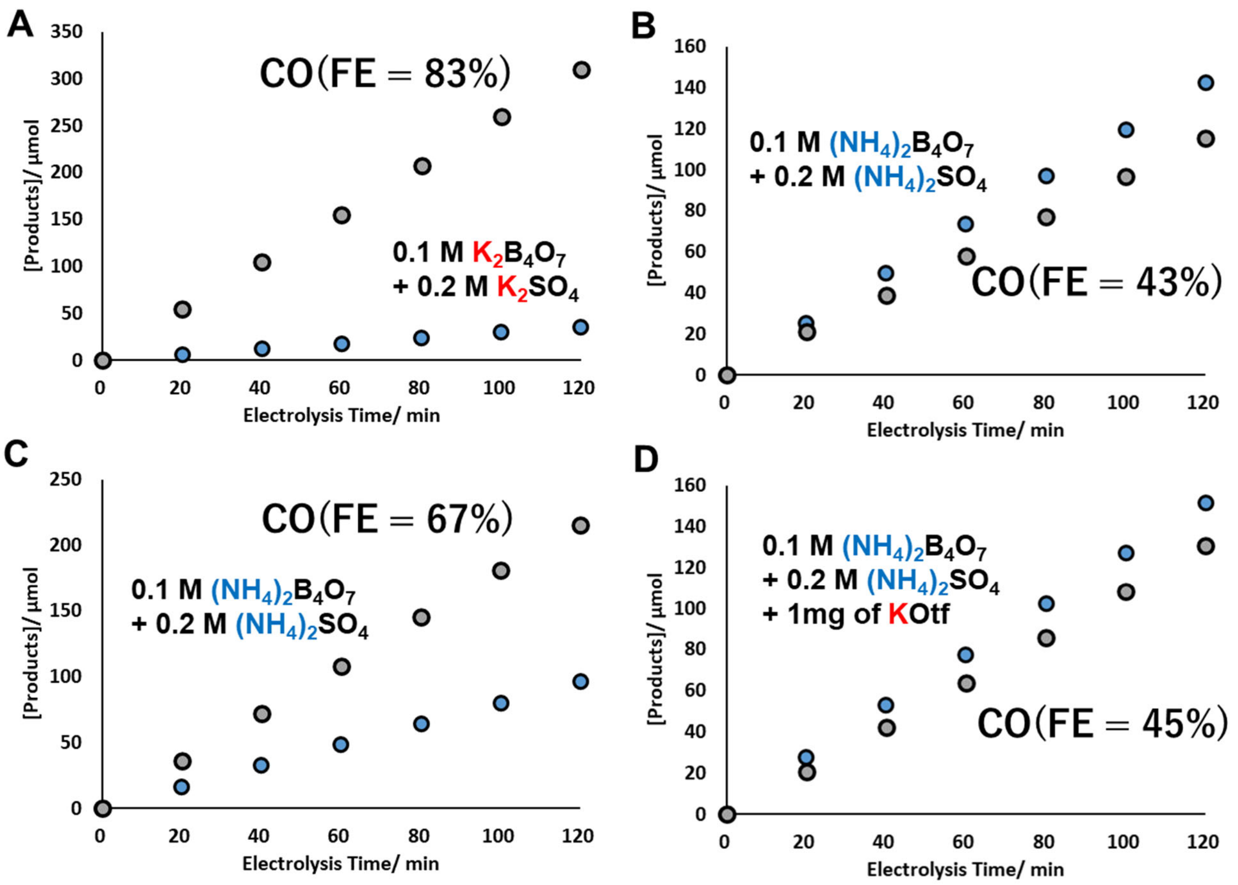

Fig. S18. Electrocatalytic activity of the Co(PyPc) electrode using $K_2B_4O_7$+K_2SO_4 or $(NH_4)_2B_4O_7$+$(NH_4)_2SO_4$ aqueous solution at -1.2 V (vs. Ag/AgCl). (A) Moles of CO (black) and H_2 (blue) produced using the Co(PyPc) electrode in $K_2B_4O_7$+K_2SO_4 aqueous solution. (B) Moles of CO (black) and H_2 (blue) produced using the Co(PyPc) electrode in $(NH_4)_2B_4O_7$+$(NH_4)_2SO_4$ aqueous solution. (C) Moles of CO (black) and H_2 (blue) produced using the Co(PyPc) with KOtf electrode in $(NH_4)_2B_4O_7$+$(NH_4)_2SO_4$ aqueous solution. (D) Moles of CO (black) and H_2 (blue) produced using the Co(PyPc) electrode in adding 1mg of KOtf $(NH_4)_2B_4O_7$+$(NH_4)_2SO_4$ aqueous solution.

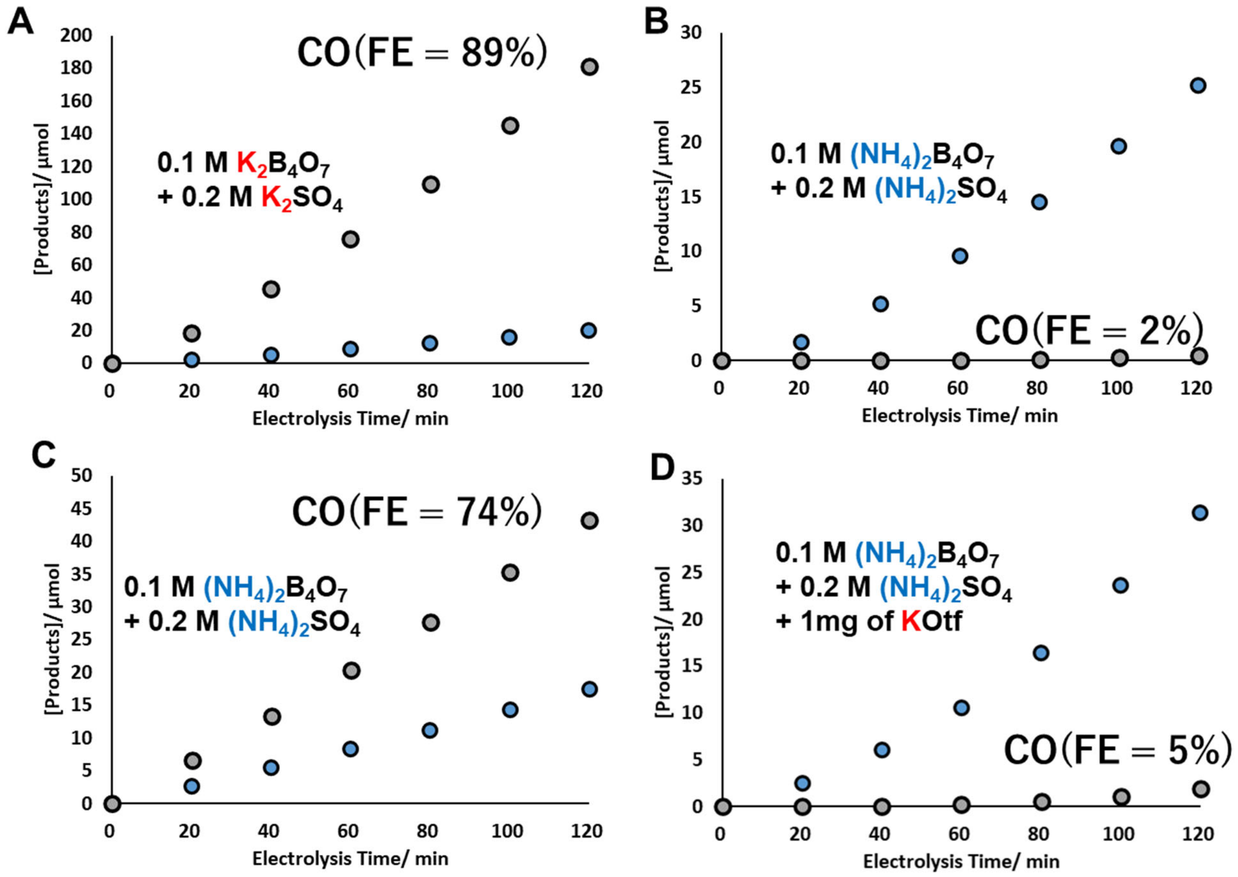

Fig. S19. Electrocatalytic activity of the Co(TPP) electrode using $K_2B_4O_7$+K_2SO_4 or $(NH_4)_2B_4O_7$+$(NH_4)_2SO_4$ aqueous solution at -1.4 V (vs. Ag/AgCl). (A) Moles of CO (black) and H_2 (blue) produced using the Co(TPP) electrode in $K_2B_4O_7$+K_2SO_4 aqueous solution. (B) Moles of CO (black) and H_2 (blue) produced using the Co(TPP) electrode in $(NH_4)_2B_4O_7$+$(NH_4)_2SO_4$ aqueous solution. (C) Moles of CO (black) and H_2 (blue) produced using the Co(TPP) with KOtf electrode in $(NH_4)_2B_4O_7$+$(NH_4)_2SO_4$ aqueous solution. (D) Moles of CO (black) and H_2 (blue) produced using the Co(TPP) electrode in adding 1mg of KOtf $(NH_4)_2B_4O_7$+$(NH_4)_2SO_4$ aqueous solution.

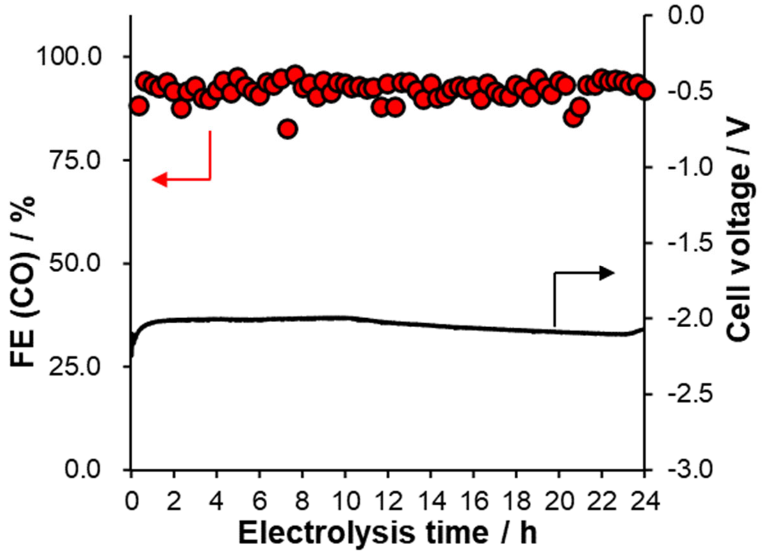

Fig. S20. Electrocatalytic CO_2 reduction using Co(Pc) catalyst. Electrocatalytic activity of the Co(Pc)+K/C electrode loaded with 0.18 mg Co(Pc) using the MEA cell during long-term bulk electrolysis at -100 mA/cm^2, as indicated by Faradaic efficiency for CO production (FE(CO), red circles) and cell voltage at a constant current density of -100 mA/cm^2 (black line).

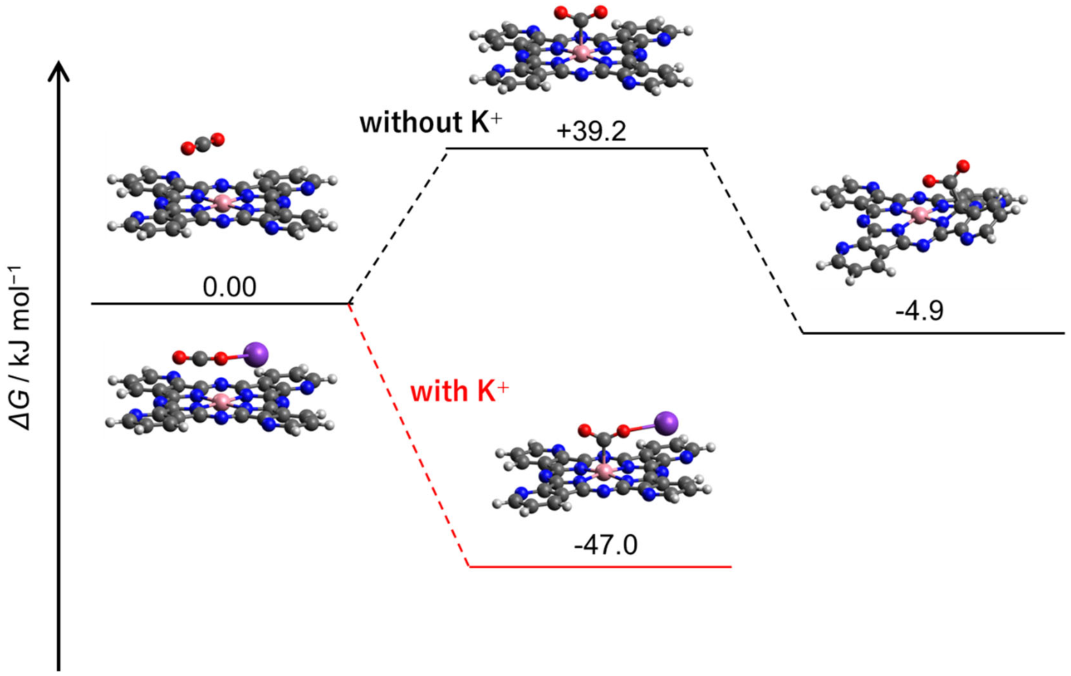

Fig. S21. DFT calculations results of Co(PyPc). Results of DFT calculations of free energy changes during catalytic reaction steps involving the two-electron reduction species with CO_2 addition to the Co(PyPc) catalyst (elements color show White (Hydrogen), Gray (Carbon), Blue (Nitrogen), Red (Oxygen), Purple (Potassium) and Pink (Cobalt)).

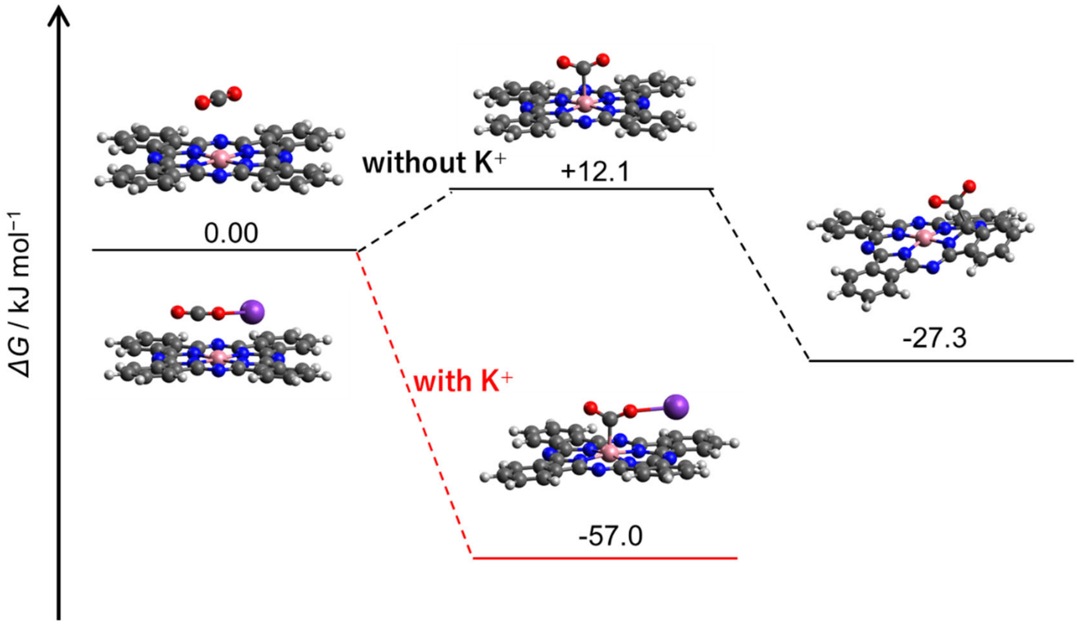

Fig. S22. DFT calculations results of Co(Pc). Results of DFT calculations of free energy changes during catalytic reaction steps involving the two-electron reduction species with CO_2 addition to the Co(Pc) catalyst (elements color show White (Hydrogen), Gray (Carbon), Blue (Nitrogen), Red (Oxygen), Purple (Potassium) and Pink (Cobalt)).

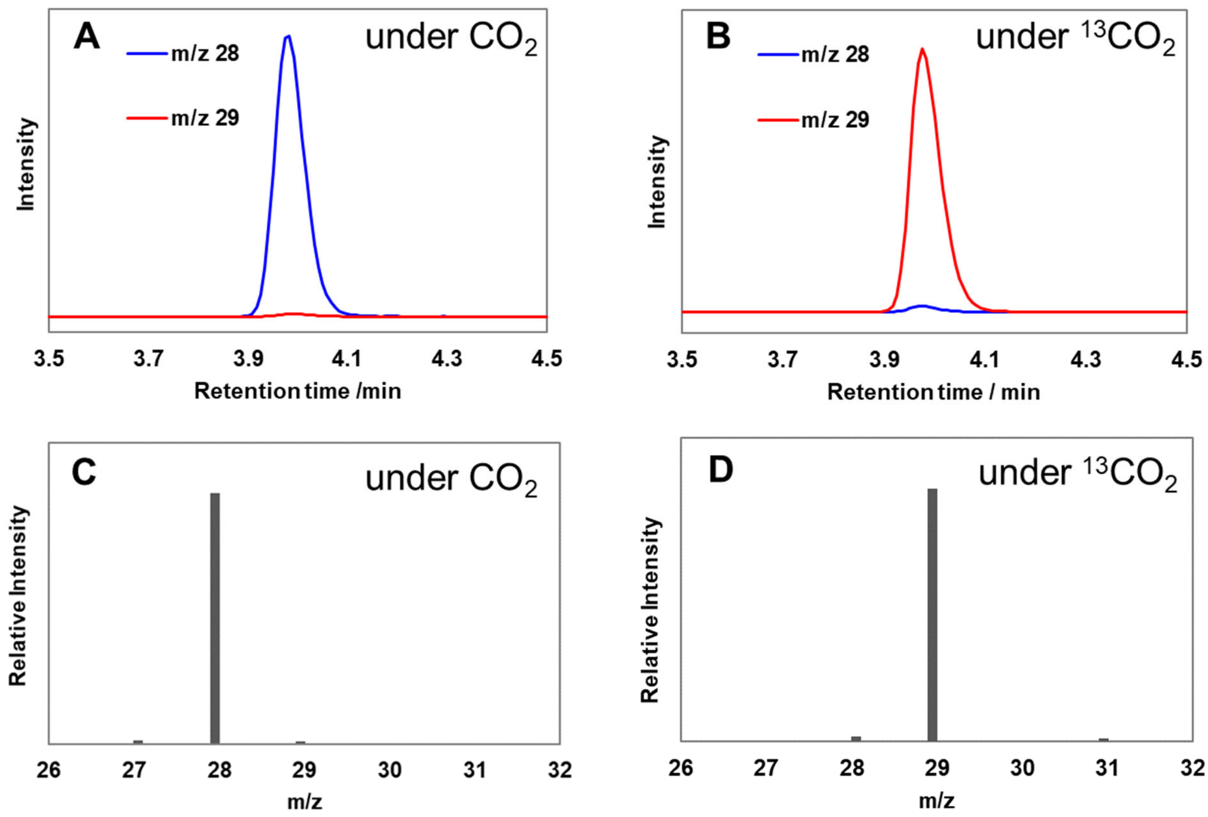

Fig. S23. Isotope experimental results for CO_2 reduction. Data obtained during isotope tracer experiments involving electrochemical CO_2 reduction using the MEA cell with the Co(PyPc)+K/C cathode and Ni+Fe/Ni anode at -1.7 V with a flow of CO_2 or $^{13}CO_2$ for 20 min. GC-MS chromatograms acquired under (A) CO_2 and (B) $^{13}CO_2$, and mass spectra acquired at a retention time of 4 min under (C) CO_2 and (D) $^{13}CO_2$.

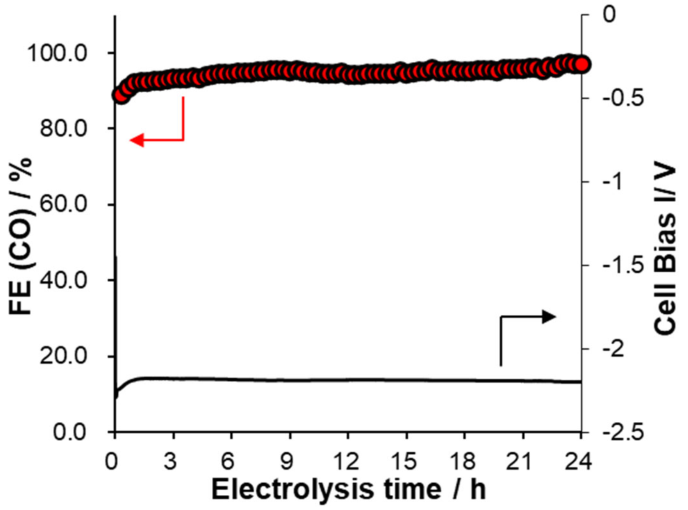

Fig. S24. Electrocatalytic CO_2 reduction using a $KHCO_3$ solution. Electrocatalytic activity of the Co(PyPc)+K/C electrode loaded with 0.24 mg Co(PyPc) using the MEA cell during long-term bulk electrolysis at -50 mA/cm^2 as indicated by the Faradaic efficiency associated with CO production (FE(CO), red circles) and cell voltage at a constant current density of -50 mA/cm^2 (black line) using 0.2 M $KHCO_3$.

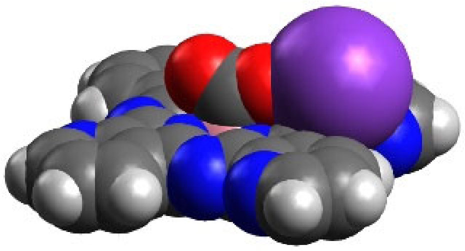

Fig. S25. Optimized molecular structure of [Co(PyPc)] with K and CO_2. Corey-Paulling-Koltun (CPK) space-filling model of the optimized molecular structure of $[Co(PyPc)(CO_2)K]^{2-}$.

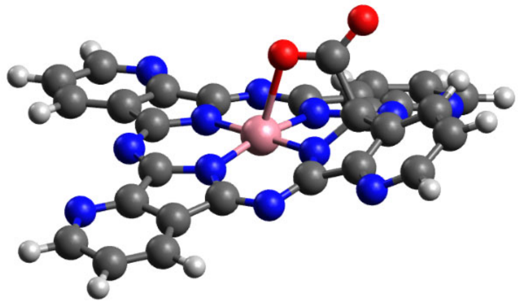

Fig. S26. Optimized molecular structure of off-center binding of CO_2 and Co(PyPc). Optimized molecular structure of $[Co(PyPc)\{OC(=O)\}]^{2-}$.

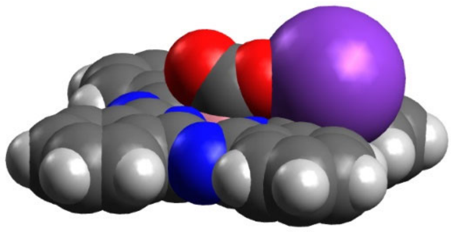

Fig. S27. Optimized molecular structure of [Co(Pc)] with K and CO_2. Corey-Paulling-Koltun (CPK) space-filling model of the optimized molecular structure of $[Co(Pc)(CO_2)K]^{2-}$.

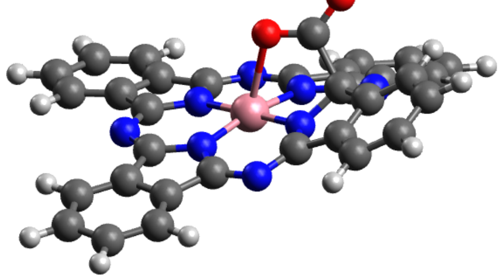

Fig. S28. Optimized molecular structure of off-center binding of CO_2 and Co(Pc). Optimized molecular structure of $[Co(Pc)\{OC(=O)\}]^{2-}$.

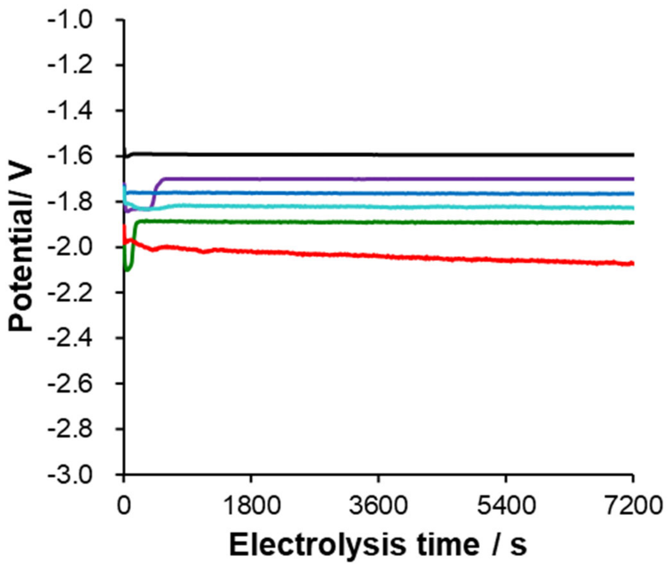

Figure S29. Applied potentials as a function of current density (-10 to -200 mA/cm^2). Chronopotentiometry data obtained during the reduction of CO_2 with 0.08 mg Co(PyPc)+K/C during 2 h of electrolysis using the MEA cell at several constant current densities [-10 mA/cm^2 (black), -25 mA/cm^2 (purple), -50 mA/cm^2 (blue), -75 mA/cm^2 (sky blue), -100 mA/cm^2 (green) and -150 mA/cm^2 (red)]. The resulting products are shown in Table 1.

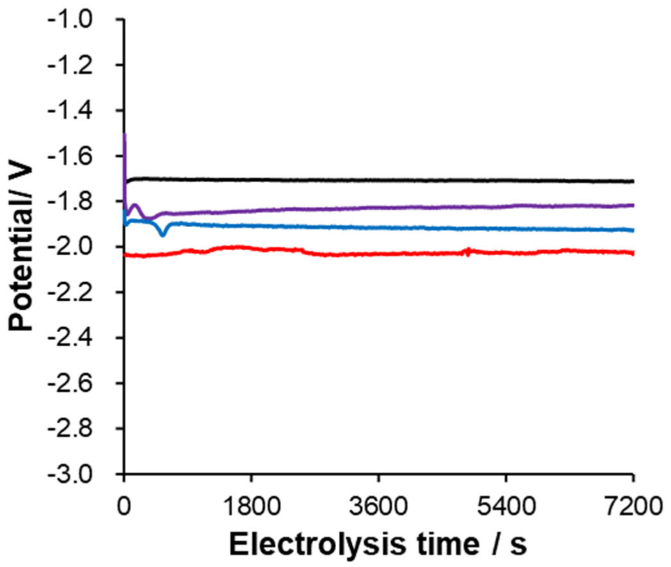

Figure S30. Applied potentials as a function of current density (-50 to -200 mA/cm^2). Chronopotentiometry data obtained employing a 0.24 mg/cm^2 Co(PyPc)+K/C electrode to catalyze CO_2 reduction over a 2 h time span using the MEA cell at several constant current densities [-50 mA/cm^2 (black), -100 mA/cm^2 (purple), -150 mA/cm^2 (blue) and -200 mA/cm^2 (red)]. The resulting products are summarized in Table 1.

Table S1. Comparison of electrocatalytic CO_2 reduction performances. Summary of electrocatalytic CO_2 reduction using full-cell gas diffusion system.

Cathode	Anode	Cell voltage / V	Current density / mA cm^{-2}	Product (FE%)	Mass activity for CO /mA mg^{-1}	Operation time / h	Reference
Co(PyPc)+K/C	Ni-Fe/Ni	-1.59	10	CO (91 %)	151.3	2	this work
Co(PyPc)+K/C	Ni-Fe/Ni	-1.95	100	CO (95 %)	1636.7	168	this work
Co(PyPc)*+K/C	Ni-Fe/Ni	-1.75	50	CO (98 %)	204.6	504	this work
Co(PyPc)*+K/C	Ni-Fe/Ni	-2.03	200	CO (95 %)	793.3	2	this work
Co(Pc)+K/C	NF	-2.1	100	CO (94 %)	522.2	24	this work
Co(Pc)+phenol	NF	ca. -2.3	100	CO (~98 %)	24.5	3	(14)
CoTMAPc@CNT	NF	ca. -1.9**	31	CO (94.5%)	430.8	15	(16)
CoPc-CN/CNT	CoOx/CNT	-2.0	ca. 37.5	CO (90%)	ca. 182	10	(15)
CoPc/CNT-MD	IrTaOx/Ti	-3.0	50	CO (90 to 80%)	ca. 5880	38	(47)
NiPcP	NF	-2.6	80	CO (no data)	-	10	(48)
Au	IrOx/Ti	-2.00	100.55	CO (98.2%)	548.8	8	(9)
Au	IrOx/Ti	-2.25	185.84	CO (85.0%)	877.5	0.67	(9)
Ag	IrOx	-3.0	50	CO (95 %)	47.5	4380	(31)
Ag	IrOx	-2.75	100	CO (95 %)	190	30	(49)
Ag	NiFeS@NF	-2.0	100	CO (98.7 %)	20	0.28	(50)
Ag	NiFeDAT	-2.18	100	CO (95 %)	100	0.05	(12)
Ag	IrOx	-2.5	230	CO (101 %)	115	0.067	(8)
AgAu	IrOx	-3.7	202	CO (93 %)	101	70	(51)

* In these trials, 0.24 mg Co(PyPc) was loaded on the electrode. ** Electrode voltage with iR compensation.

CNT = carbon nanotube, NF = Ni-foam

Table S2. Comparison of electrocatalytic CO_2 reduction performances using various additives. Summary of electrocatalytic CO_2 reduction trials using Co complex catalysts with various additives in full-cell gas diffusion systems.

Cathode catalyst	Anode catalyst	Additives	Cell voltage / V	Current density / mA cm^{-2}	Product (FE%)	Operation time / h	Reference
Co(PyPc)	Ni-Fe/Ni	KOtf	-1.95	100	CO (95 %)	168	this work
Co(PyPc)	Ni-Fe/Ni	KC_4F_9Otf	-1.90	100	CO (95 %)	24	this work
Co(PyPc)	Ni-Fe/Ni	NaOtf	-1.86	50	CO (93 %)	24	this work
Co(PyPc)	Ni-Fe/Ni	K_2CO_3	-2.0	50	CO (95 to 65 %)	4	this work
Co(PyPc)	Ni-Fe/Ni	K (Tf_2N)	-1.89	100	CO (94 %)	24	this work
Co(PyPc)	Ni-Fe/Ni	Mg $(Tf_2N)_2$	-1.77	50	CO (88 %)	24	this work
Co(PyPc)	Ni-Fe/Ni	Mg $(Tf_2N)_2$	-1.95	100	CO (80 %)	24	this work
Co(PyPc)	Ni-Fe/Ni	Cs (Tf_2N)	-1.97	50	CO (94 %)	24	this work
Co(PyPc)	Ni-Fe/Ni	KOH	-*	100	-*	Less than 2h	this work
Co(PyPc)	Ni-Fe/Ni	-	-2.1	100	CO (94 to 47 %)	4	this work
Co(Pc)	Ni-foam	KOtf	-2.1	100	CO (94 %)	24	this work
Co(Pc)	Ni-foam	phenol	ca. -2.3	100	CO (98 to ca.75 %)	4	(14)
Co(Pc)	Ni-foam	-	ca. -2.7	100	CO (98 to ca. 65 %)	4	(14)

*Due to salt precipitation, we could not measure the production of CO and H_2 correctly.

Table S3. Comparison of electrocatalytic CO_2 reduction performances using $KHCO_3$ solution. Summary of electrocatalytic CO_2 reduction using full-cell gas diffusion system using carbonate salt solution.

Cathode catalyst	Anode catalyst	Solvent	Cell voltage / V	Current density / mA cm^{-2}	Product (FE%)	Operation time / h	Reference
**Co(PyPc) +K**	**IrOx**	**0.2M $KHCO_3$**	**-2.2**	**50**	**CO (94 %)**	**24**	**this work**
**CoPc/CNT -MD**	**IrTaOx/Ti**	**0.5 M $KHCO_3$**	**-3.0**	**50**	**CO (90 to 80%)**	**38**	**(47)**
**Ag**	**IrOx**	**0.01 M $KHCO_3$**	**-3.0**	**50**	**CO (95 %)**	**4380**	**(31)**
**AgAu**	**IrOx**	**1M $KHCO_3$**	**~-4.0**	**100**	**CO (80 %)**	**-**	**(51)**

Table S4 Summarized DFT results of Co(PyPc) and Co(Pc). Calculated Gibbs free energies (*G*) and ΔG_{ads} values for CO_2 adsorption reactions.

reaction	Co(PyPc)				Co(Pc)			
	G (hartree)		ΔG_{ads}		*G* (hartree)		ΔG_{ads}	
	before adsorption	after adsorption	(hartree)	(kJ/mol)	before adsorption	after adsorption	(hartree)	(kJ/mol)
(1)	−2064.416504	−2064.401577	+0.014927	+39.2	−2000.241082	−2000.236481	+0.004601	+12.1
(2)	−2664.356214	−2664.374088	−0.017874	−46.9	−2600.177695	−2600.200102	−0.022407	−58.8
(3)	−2064.416504	−2064.418383	−0.001879	−4.9	−2000.241082	−2000.251499	+0.010417	−27.3

REFERENCES AND NOTES

1. W. H. Cheng, M. H. Richter, I. Sullivan, D. M. Larson, C. X. Xiang, B. S. Brunschwig, H. A. Atwater, CO_2 reduction to CO with 19% efficiency in a solar-driven gas diffusion electrode flow cell under outdoor solar illumination. *Acs Energy Lett.* **5**, 470–476 (2020).

2. Y. Xiao, Y. Qian, A. Chen, T. Qin, F. Zhang, H. Tang, Z. Qiu, B.-L. Lin, An artificial photosynthetic system with CO_2-reducing solar-to-fuel efficiency exceeding 20%. *J. Mater. Chem. A* **8**, 18310–18317 (2020).

3. H. A. Schwarz, R. W. Dodson, Reduction potentials of CO_2^- and the alcohol radicals. *J. Phys. Chem.* **93**, 409–414 (1989).

4. A. J. Morris, G. J. Meyer, E. Fujita, Molecular approaches to the photocatalytic reduction of carbon dioxide for solar fuels. *Acc. Chem. Res.* **42**, 1983–1994 (2009).

5. Y. Hori, K. Kikuchi, S. Suzuki, Production of CO and CH_4 in electrochemical reduction of CO_2 at metal electrodes in aqueous hydrogencarbonate solution. *Chem. Lett.* **14**, 1695–1698 (1985).

6. P. De Luna, C. Hahn, D. Higgins, S. A. Jaffer, T. F. Jaramillo, E. H. Sargent, What would it take for renewably powered electrosynthesis to displace petrochemical processes? *Science* **364**, eaav3506 (2019).

7. S. Nitopi, E. Bertheussen, S. B. Scott, X. Liu, A. K. Engstfeld, S. Horch, B. Seger, I. E. L. Stephens, K. Chan, C. Hahn, J. K. Norskov, T. F. Jaramillo, I. Chorkendorff, Progress and perspectives of electrochemical CO_2 reduction on copper in aqueous electrolyte. *Chem. Rev.* **119**, 7610–7672 (2019).

8. S. Verma, X. Lu, S. C. Ma, R. I. Masel, P. J. A. Kenis, The effect of electrolyte composition on the electroreduction of CO_2 to CO on Ag based gas diffusion electrodes. *Phys. Chem. Chem. Phys.* **18**, 7075–7084 (2016).

9. S. Verma, Y. Hamasaki, C. Kim, W. X. Huang, S. Lu, H. R. M. Jhong, A. A. Gewirth, T. Fujigaya, N. Nakashima, P. J. A. Kenis, Insights into the low overpotential electroreduction of CO_2 to Co on a supported gold catalyst in an alkaline flow electrolyzer. *Acs Energy Lett.* **3**, 193–198 (2018).

10. F. P. G. de Arquer, C. T. Dinh, A. Ozden, J. Wicks, C. McCallum, A. R. Kirmani, D. H. Nam, C. Gabardo, A. Seifitokaldani, X. Wang, Y. G. C. Li, F. W. Li, J. Edwards, L. J. Richter, S. J. Thorpe, D. Sinton, E. H. Sargent, CO_2 electrolysis to multicarbon products at activities greater than 1 A cm^{-2}. *Science* **367**, 661–666 (2020).

11. R. I. Masel, Z. Liu, H. Yang, J. J. Kaczur, D. Carrillo, S. Ren, D. Salvatore, C. P. Berlinguette, An industrial perspective on catalysts for low-temperature CO_2 electrolysis. *Nat. Nanotechnol.* **16**, 118–128 (2021).

12. S. S. Bhargava, D. Azmoodeh, X. Chen, E. R. Cofell, A. M. Esposito, S. Verma, A. A. Gewirth, P. J. A. Kenis, Decreasing the energy consumption of the CO_2 electrolysis process using a magnetic field. *ACS Energy Lett.* **6**, 2427–2433 (2021).

13. D. Wakerley, S. Lamaison, J. Wicks, A. Clemens, J. Feaster, D. Corral, S. A. Jaffer, A. Sarkar, M. Fontecave, E. B. Duoss, S. Baker, E. H. Sargent, T. F. Jaramillo, C. Hahn, Gas diffusion electrodes, reactor designs and key metrics of low-temperature CO_2 electrolysers. *Nat. Energy* **7**, 130–143 (2022).

14. S. Ren, D. Joulie, D. Salvatore, K. Torbensen, M. Wang, M. Robert, C. P. Berlinguette, Molecular electrocatalysts can mediate fast, selective CO_2 reduction in a flow cell. *Science* **365**, 367–369 (2019).

15. X. Lu, Y. S. Wu, X. L. Yuan, L. Huang, Z. S. Wu, J. Xuan, Y. F. Wang, H. L. Wang, High-performance electrochemical CO_2 reduction cells based on non-noble metal catalysts. *Acs Energy Lett.* **3**, 2527–2532 (2018).

16. J. Su, J.-J. Zhang, J. Chen, Y. Song, L. Huang, M. Zhu, B. I. Yakobson, B. Z. Tang, R. Ye, Building a stable cationic molecule/electrode interface for highly efficient and durable CO_2 reduction at an industrially relevant current. *Energ. Environ. Sci.* **14**, 483–492 (2021).

17. X. Zhang, Y. Wang, M. Gu, M. Wang, Z. Zhang, W. Pan, Z. Jiang, H. Zheng, M. Lucero, H. Wang, G. E. Sterbinsky, Q. Ma, Y.-G. Wang, Z. Feng, J. Li, H. Dai, Y. Liang, Molecular engineering of dispersed nickel phthalocyanines on carbon nanotubes for selective CO_2 reduction. *Nat. Energy* **5**, 684–692 (2020).

18. B. Siritanaratkul, M. Forster, F. Greenwell, P. K. Sharma, E. H. Yu, A. J. Cowan, Zero-gap bipolar membrane electrolyzer for carbon dioxide reduction using acid-tolerant molecular electrocatalysts. *J. Am. Chem. Soc.* **144**, 7551–7556 (2022).

19. H. Takeda, C. Cometto, O. Ishitani, M. Robert, Electrons, photons, protons and earth-abundant metal complexes for molecular catalysis of CO_2 reduction. *ACS Catal.* **7**, 70–88 (2017).

20. S. Sato, K. Saita, K. Sekizawa, S. Maeda, T. Morikawa, Low-energy electrocatalytic CO_2 reduction in water over Mn-complex catalyst electrode aided by a nanocarbon support and K^+ cations. *ACS Catal.* **8**, 4452–4458 (2018).

21. E. Villagra, F. Bedioui, T. Nyokong, J. C. Canales, M. Sancy, M. A. Páez, J. Costamagna, J. H. Zagal, Tuning the redox properties of Co-N4 macrocyclic complexes for the catalytic electrooxidation of glucose. *Electrochim. Acta* **53**, 4883–4888 (2008).

22. C. Linares-Flores, D. M. Carey, A. Munoz-Castro, J. H. Zagal, J. Pavez, D. Pino-Riffo, R. Arratia-Perez, Reinterpreting the role of the catalyst formal potential. The case of thiocyanate electrooxidation catalyzed by CoN4-macrocyclic complexes. *J. Phys. Chem. C* **116**, 7091–7098 (2012).

23. K. Koike, H. Hori, M. Ishizuka, J. R. Westwell, K. Takeuchi, T. Ibusuki, K. Enjouji, H. Konno, K. Sakamoto, O. Ishitani, Key process of the photocatalytic reduction of CO_2 using $[Re(4,4'\text{-}X_2\text{-bipyridine})(CO)_3PR_3]^+$(X = CH_3, H, CF_3; PR_3= Phosphorus Ligands): Dark

reaction of the one-electron-reduced complexes with CO_2. *Organometallics* **16**, 5724–5729 (1997).

24. T. M. Suzuki, T. Nonaka, K. Kitazumi, N. Takahashi, S. Kosaka, Y. Matsuoka, K. Sekizawa, A. Suda, T. Morikawa, Highly enhanced electrochemical water oxidation reaction over hyperfine β-FeOOH(Cl):Ni nanorod electrode by modification with amorphous $Ni(OH)_2$. *Bull. Chem. Soc. Jpn.* **91**, 778–786 (2018).

25. T. Arai, S. Sato, K. Sekizawa, T. M. Suzuki, T. Morikawa, Solar-driven CO_2 to CO reduction utilizing H_2O as an electron donor by earth-abundant Mn-bipyridine complex and Ni-modified Fe-oxyhydroxide catalysts activated in a single-compartment reactor. *Chem. Commun.* **55**, 237–240 (2019).

26. L.-C. Weng, A. T. Bell, A. Z. Weber, Towards membrane-electrode assembly systems for CO_2 reduction: A modeling study. *Energ. Environ. Sci.* **12**, 1950–1968 (2019).

27. D. G. Wheeler, B. A. W. Mowbray, A. Reyes, F. Habibzadeh, J. He, C. P. Berlinguette, Quantification of water transport in a CO_2 electrolyzer. *Energ. Environ. Sci.* **13**, 5126–5134 (2020).

28. L. Lin, T. Liu, J. Xiao, H. Li, P. Wei, D. Gao, B. Nan, R. Si, G. Wang, X. Bao, Enhancing CO_2 electroreduction to methane with a cobalt phthalocyanine and zinc-nitrogen-carbon tandem catalyst. *Angew. Chem. Int. Ed. Engl.* **59**, 22408–22413 (2020).

29. A. N. Marianov, A. S. Kochubei, T. Roman, O. J. Conquest, C. Stampfl, Y. Jiang, Resolving deactivation pathways of Co porphyrin-based electrocatalysts for CO_2 reduction in aqueous medium. *ACS Catal.* **11**, 3715–3729 (2021).

30. Y. Dong, C. W. Oloman, E. L. Gyenge, J. Su, L. Chen, Transition metal based heterogeneous electrocatalysts for the oxygen evolution reaction at near-neutral pH. *Nanoscale* **12**, 9924–9934 (2020).

31. R. B. Kutz, Q. Chen, H. Yang, S. D. Sajjad, Z. Liu, I. R. Masel, Sustainion imidazolium-functionalized polymers for carbon dioxide electrolysis. *Energ. Technol.* **5**, 929–936 (2017).

32. J. D. Chai, M. Head-Gordon, Long-range corrected hybrid density functionals with damped atom-atom dispersion corrections. *Phys. Chem. Chem. Phys.* **10**, 6615–6620 (2008).

33. W. J. Stevens, M. Krauss, H. Basch, P. G. Jasien, Relativistic compact effective potentials and efficient, shared-exponent basis sets for the third-, fourth-, and fifth-row atoms. *Can. J. Chem.* **70**, 612–630 (1992).

34. V. A. Rassolov, M. A. Ratner, J. A. Pople, P. C. Redfern, L. A. Curtiss, 6-31G* basis set for third-row atoms. *J. Comput. Chem.* **22**, 976–984 (2001).

35. P. C. Hariharan, J. A. Pople, The influence of polarization functions on molecular orbital hydrogenation energies. *Theor. Chem. Acc* **28**, 213–222 (1973).

36. W. J. Hehre, R. Ditchfield, J. A. Pople, Self-consistent molecular orbital methods. XII. Further Extensions of Gaussian-type basis sets for use in molecular orbital studies of organic molecules. *J. Chem. Phys.* **56**, 2257–2261 (1972).

37. R. Ditchfield, W. J. Hehre, J. A. Pople, Self-consistent molecular-orbital methods. IX. An extended Gaussian-type basis for molecular-orbital studies of organic molecules. *J. Chem. Phys.* **54**, 724–728 (1971).

38. G. Scalmani, M. J. Frisch, Continuous surface charge polarizable continuum models of solvation. I. General formalism. *J. Chem. Phys.* **132**, 114110 (2010).

39. J. Tomasi, B. Mennucci, R. Cammi, Quantum mechanical continuum solvation models. *Chem. Rev.* **105**, 2999–3094 (2005).

40. J. Tomasi, B. Mennucci, E. Cances, The IEF version of the PCM solvation method: An overview of a new method addressed to study molecular solutes at the QM ab initio level. *J. Mol. Struc.: THEOCHEM* **464**, 211–226 (1999).

41. B. Mennucci, J. Tomasi, Continuum solvation models: A new approach to the problem of solute's charge distribution and cavity boundaries. *J. Chem. Phys.* **106**, 5151–5158 (1997).

42. B. Mennucci, E. Cances, J. Tomasi, Evaluation of solvent effects in isotropic and anisotropic dielectrics and in ionic solutions with a unified integral equation method: Theoretical bases, computational implementation, and numerical applications. *J. Phys. Chem. B* **101**, 10506–10517 (1997).

43. E. Cances, B. Mennucci, J. Tomasi, A new integral equation formalism for the polarizable continuum model: Theoretical background and applications to isotropic and anisotropic dielectrics. *J. Chem. Phys.* **107**, 3032–3041 (1997).

44. M. J. Frisch, G. W. Trucks, H. B. Schlegel, G. E. Scuseria, M. A. Robb, J. R. Cheeseman, G. Scalmani, V. Barone, G. A. Petersson, H. Nakatsuji, X. Li, M. Caricato, A. V. Marenich, J. Bloino, B. G. Janesko, R. Gomperts, B. Mennucci, H. P. Hratchian, J. V. Ortiz, A. F. Izmaylov, J. L. Sonnenberg, Williams, F. Ding, F. Lipparini, F. Egidi, J. Goings, B. Peng, A. Petrone, T. Henderson, D. Ranasinghe, V. G. Zakrzewski, J. Gao, N. Rega, G. Zheng, W. Liang, M. Hada, M. Ehara, K. Toyota, R. Fukuda, J. Hasegawa, M. Ishida, T. Nakajima, Y. Honda, O. Kitao, H. Nakai, T. Vreven, K. Throssell, J. A. Montgomery Jr., J. E. Peralta, F. Ogliaro, M. J. Bearpark, J. J. Heyd, E. N. Brothers, K. N. Kudin, V. N. Staroverov, T. A. Keith, R. Kobayashi, J. Normand, K. Raghavachari, A. P. Rendell, J. C. Burant, S. S. Iyengar, J. Tomasi, M. Cossi, J. M. Millam, M. Klene, C. Adamo, R. Cammi, J. W. Ochterski, R. L. Martin, K. Morokuma, O. Farkas, J. B. Foresman, D. J. Fox, Gaussian 16, Revision C.01, Gaussian, Inc., Wallingford CT (2016).

45. R. Shannon, Revised effective ionic radii and systematic studies of interatomic distances in halides and chalcogenides. *Acta Crystallogr. A* **32**, 751–767 (1976).

46. T. Nonaka, K. Dohmae, T. Araki, Y. Hayashi, Y. Hirose, T. Uruga, H. Yamazaki, T. Mochizuki, H. Tanida, S. Goto, Quick-scanning x-ray absorption spectroscopy system with a servo-motor-driven channel-cut monochromator with a temporal resolution of 10 ms. *Rev. Sci. Instrum.* **83**, 083112 (2012).

47. X. Wu, J. W. Sun, P. F. Liu, J. Y. Zhao, Y. Liu, L. Guo, S. Dai, H. G. Yang, H. Zhao, Molecularly dispersed cobalt phthalocyanine mediates selective and durable CO_2 reduction in a membrane flow cell. *Adv. Funct. Mater.* **32**, 2107301 (2022).

48. S. Wei, H. Zou, W. Rong, F. Zhang, Y. Ji, L. Duan, Conjugated nickel phthalocyanine polymer selectively catalyzes CO_2-to-CO conversion in a wide operating potential window. *Appl. Catal. Environ.* **284**, 119739 (2021).

49. W. H. Lee, Y.-J. Ko, Y. Choi, S. Y. Lee, C. H. Choi, Y. J. Hwang, B. K. Min, P. Strasser, H.-S. Oh, Highly selective and scalable CO_2 to CO-electrolysis using coral-nanostructured Ag catalysts in zero-gap configuration. *Nano Energy* **76**, 105030 (2020).

50. S. Li, Y. Ma, T. Zhao, J. Li, X. Kang, W. Guo, Y. Wen, L. Wang, Y. Wang, R. Lin, T. Li, H. Tan, H. Peng, B. Zhang, Polymer-supported liquid layer electrolyzer enabled electrochemical CO_2 reduction to CO with high energy efficiency. *ChemistryOpen* **10**, 639–644 (2021).

51. J. Li, A. Zitolo, F. A. Garcés-Pineda, T. Asset, M. Kodali, P. Tang, J. Arbiol, J. R. Galán-Mascarós, P. Atanassov, I. V. Zenyuk, M. T. Sougrati, F. Jaouen, Metal oxide clusters on nitrogen-doped carb on are highly selective for CO_2 electroreduction to CO. *ACS Catal.* **11**, 10028–10042 (2021).
